# Similar immune responses to alpha1‐oleate and Bacillus Calmette–Guérin treatment in patients with bladder cancer

**DOI:** 10.1002/cam4.7091

**Published:** 2024-03-29

**Authors:** Shahram Ahmadi, Ines Ambite, Antonín Brisuda, Jaromír Háček, Farhan Haq, Samudra Sabari, Kamala Vanarsa, Chandra Mohan, Marek Babjuk, Catharina Svanborg

**Affiliations:** ^1^ Division of Microbiology, Immunology and Glycobiology, Department of Laboratory Medicine, Faculty of Medicine Lund University Lund Sweden; ^2^ Department of Urology Motol University Hospital, 2nd Faculty of Medicine, Charles University Praha Prague Czech Republic; ^3^ Department of Pathology and Molecular Medicine Motol University Hospital, 2nd Faculty of Medicine, Charles University Praha Prague Czech Republic; ^4^ Department of Biomedical Engineering University of Houston Houston Texas USA

**Keywords:** alpha1‐oleate, BCG, bladder cancer, immune response, proteomic analysis

## Abstract

**Background:**

The molecular content of urine is defined by filtration in the kidneys and by local release from tissues lining the urinary tract. Pathological processes and different therapies change the molecular composition of urine and a variety of markers have been analyzed in patients with bladder cancer. The response to BCG immunotherapy and chemotherapy has been extensively studied and elevated urine concentrations of IL‐1RA, IFN‐α, IFN‐γ TNF‐α, and IL‐17 have been associated with improved outcome.

**Methods:**

In this study, the host response to intravesical alpha 1‐oleate treatment was characterized in patients with non‐muscle invasive bladder cancer by proteomic and transcriptomic analysis.

**Results:**

Proteomic profiling detected a significant increase in multiple cytokines in the treatment group compared to placebo. The innate immune response was strongly activated, including IL‐1RA and pro‐inflammatory cytokines in the IL‐1 family (IL‐1α, IL‐1β, IL‐33), chemokines (MIP‐1α, IL‐8), and interferons (IFN‐α2, IFN‐γ). Adaptive immune mediators included IL‐12, Granzyme B, CD40, PD‐L1, and IL‐17D, suggesting broad effects of alpha 1‐oleate treatment on the tumor tissues.

**Conclusions:**

The cytokine response profile in alpha 1‐oleate treated patients was similar to that reported in BCG treated patients, suggesting a significant overlap. A reduction in protein levels at the end of treatment coincided with inhibition of cancer‐related gene expression in tissue biopsies, consistent with a positive treatment effect. Thus, in addition to killing tumor cells and inducing cell detachment, alpha 1‐oleate is shown to activate a broad immune response with a protective potential.

## 1 INTRODUCTION

Bladder cancer is the fourth most common malignancy in the United States and the fifth in Europe, with a prevalence of about 1/4,000.^1^ Each year, around 500,000 patients worldwide are diagnosed and this number is rising.^2^ Furthermore, bladder cancer, with 70%–80% of cases presenting as non‐muscle invasive, has the highest recurrence rate of all cancer indications as more than 80% recur after complete surgical removal of the first tumor and 15% progress to muscle invasive disease.^3^ Because of the absence of curative treatments for systemic disease around 165,000 deaths are registered each year, world‐wide.^2^ Due to high recurrence rates and a lack of curative therapies, bladder cancer has the highest lifetime treatment cost per patient of all cancers, followed by colorectal, breast, prostate, and lung cancers.^2^, ^4^ Chemotherapy and immunotherapy are often suboptimal due to significant side effects and limited efficacy.

The alpha1‐oleate complex kills bladder cancer cells and has shown potent therapeutic effects in a murine bladder cancer model.^5^ A recent, randomized, placebo‐controlled study of patients with non‐muscle invasive bladder cancer (NMIBC) showed a significant treatment effect of repeated intravesical instillations, with a reduction in tumor size.^6^ Alpha1‐oleate instillations triggered a rapid tumor response, quantified as the shedding of tumor cells into the urine within 2 h of treatment. Tumor cell death was accompanied by a strong apoptosis‐like response in the tumor, and shed cells contained large amounts of alpha1‐oleate. Sequencing of tumor RNA revealed major inhibitory effects of alpha1‐oleate on cancer related gene expression, identifying potent effects of the peptide‐lipid complex on bladder cancer tissue.^6^

Proteomic and genomic analyses have been extensively used to follow treatment responses and predict the clinical outcomes of different cancer therapies.^7^, ^8^ Nuclear matrix protein 22 (NMP22 or NuMA) and human complement factor H related protein (hCFHrp) are two Food and Drug Administration (FDA) approved biomarkers for bladder cancer detection with reported high sensitivity.^9^, ^10^ Similarly, the urinary bladder cancer rapid test, which detects fragments of cytokeratins 8 and 18, has shown 87% sensitivity in patients with cancer in situ, but lower sensitivity in low and high grade tumors.^11^ The studies investigating different treatment responses have predominantly included cytokines and chemokines.^12^ For example, Bacillus Calmette–Guérin (BCG) treatment activates an innate immune response including the interleukin (IL)‐1 family of cytokines, as well as IL‐6, IL‐8, IFN‐γ, and TNF‐α. T‐cell responses and especially IL‐4, IL‐2, Granzyme B, IL‐12, and IL‐17 have been associated with the outcome of BCG therapy.^13^, ^14^, ^15^, ^16^, ^17^, ^18^

In this study, the response to alpha1‐oleate treatment was quantified by proteomic analysis of urine samples obtained from patients with NMIBC. The results identify a rapid innate immune response, characterized by pro‐inflammatory cytokines as well as regulators of adaptive immunity. Interestingly, there was a significant overlap between the response to alpha1‐oleate and the reported immune response to BCG therapy including IL‐1RA, IL‐1α, IL‐8, IFN‐γ, IFN‐α2, TNF‐α, Granzyme B, IL‐12 and IL‐17,^15^, ^16^, ^17^, ^19^, ^20^, ^21^, ^22^ previously correlated with a positive outcome in BCG treated patients. Moreover, a comparison of the proteomic profile with gene expression in treated tissues, revealed a change over time, from acute innate immune activation to a later reduction in cytokine levels in urine and tissue biopsies, suggesting that an initial increase, followed by a reduction in cytokine levels, may predict a beneficial treatment effect.

## 2 MATERIALS AND METHODS

### 2.1 Patient population and approval

This single‐center study (HP002‐001) included patients, who were diagnosed with NMIBC and scheduled for transurethral surgery.^6^ The study was approved by the State Institute for Drug Control (SUKL) in the Czech Republic; number 273799/17‐I and the Ethics Committee of the Motol University Hospital; number EK‐786/17 (ClinicalTrials.gov Identifier: NCT03560479). Patients gave their written informed consent. Demographic data, morbidity and health parameters as well as tumor characteristics were recorded by the study physicians in the electronic Case Report Form (eCRF) and closely monitored by an external monitor.

### 2.2 Study protocol

The first, part of the single center (EudraCTNo:2016‐004269‐14, ClinicalTrials.gov NCT03560479) was placebo‐controlled, enrolling 20 patients in the active arm and 20 patients in the placebo arm. Subjects were randomized 1/1 and received intra‐vesical instillations (30 mL) of either alpha1–oleate (1.7 mM) or Phosphate Buffered Saline (PBS) on six occasions during a 22‐day period (Days 1, 3, 5, 8, 15, and 22) (Figure S1). The inclusion criteria were as follows:
Patient with non‐muscle invasive papillary bladder cancer (NMIBC) based on cystoscopy appearance, on the waiting list for transurethral resection of bladder tumor (TURBT).
Male and female subjects, 18 years or older.
Patients should be able to keep the content of the bladder for at least 1 hour.
Signed and dated informed consent form.
Negative pregnancy test in women of childbearing potential.
Men and women of childbearing potential should use appropriate methods of contraception during the study. Men should also refrain from donating sperm.

The second, dose‐finding part of the study, used the same eligibility criteria, schedule of visits, study treatment administration, primary and secondary end points and assessments, as the first part of the study. Six patients received intra‐vesical instillations of alpha1‐oleate at a five times higher dose (8.5 mM) and three patients a 10‐times higher dose (17 mM) than patients in the first part of the study, receiving 1.7 mM (*n* = 20) or placebo (*n* = 20). The six patients receiving 8.5 mM of alpha1‐oleate completed the treatment and samples from these patients were included in this study.

### 2.3 Analysis of urine samples

Urine samples were collected from patients in the placebo‐controlled part of the study before and about 2 h after each instillation of alpha1‐oleate or placebo, on Days 1, 3, 5, 8, 15, and 22 (6 visits) and stored at −80°C. A total of 311 samples were analyzed from the 20 patients who received 1.7 mM of alpha1‐oleate, 20 who received placebo and six who received the higher dose of 8.5 mM of alpha1‐oleate. Cell numbers in uncentrifuged urine samples were used for the quantification of cell shedding by microscopy, cells harvested by cytospin onto microscope slides were used for immunohistochemistry staining for specific cytokines and to quantify the content of alpha1‐oleate in the shed tumor cells.

### 2.4 Proteomic analysis

The Luminex micro‐bead assay employs conjugated microsphere particles to immobilize specific antigens on their surface. Using dual fluorescence signals, the instrument determines the analyte attached to the bead and calculates its corresponding concentration based on the detection dye's intensity. As a result, the Luminex system enables the simultaneous detection and quantification of multiple biomarkers.

In this study, we first utilized this assay to screen for 16 potential urine biomarkers (Eotaxin, GRO, IFN‐α, IL‐1α, IL‐1RA, IL‐7, IL‐8, IL‐15, IL‐31, IP‐10, MIP‐1α, MIP‐1β, MCP‐1, RANTES, SDF‐1α, and TNF‐β), by quantifying the concentration of each marker in urine (Cat # HCYTMAG‐60K‐PX41, Lot # 3090739). Briefly, standards or urine samples were diluted 1:50, and beads were added to the wells. Following incubation, the plate underwent washing before the addition of biotinylated detection antibodies. After a second incubation step, Streptavidin‐Phycoerythrin (the detection dye) was introduced, incubated, and then washed. Lastly, sheath fluid was introduced into each well, and the plate was read, collecting 50 beads per analyte.

The analysis was subsequently extended using the Human Immunotherapy Luminex Performance Assay 24‐plex Fixed Panel to quantify CCL2/MCP‐1, CCL3/MIP‐1α, CCL4/MIP‐1β, CD40Ligand/TNFSF5, CXCL10/IP‐10, GM‐CSF, Granzyme B, IFN‐α, IFN‐γ, IL‐1α, IL‐1β, IL‐1RA, IL‐2, IL‐4, IL‐6, IL‐8/CXCL8, IL‐10, IL‐12 p70, IL‐13, IL‐15, IL‐17/IL‐17A, IL‐33, PD‐L1/B7‐H1 and TNF‐α concentrations in urine (R&D Systems, Cat # HCYTMAG‐60K‐PX41, Lot # 3090739).

After thawing on ice, urine samples were gently mixed and used undiluted. Reverse pipetting was used for high accuracy in all liquid handling steps. High and low controls, all standards and buffer controls were analyzed in duplicates. The assays were performed according to the manufacturer's instructions. Standards, low and high controls were reconstituted with diluent RD6‐65, sat for 15 min, followed by 5 min of gentle agitation. A 7 point standard curve using 3‐fold serial dilutions in, diluent RD6‐65 was used. Briefly, 50 μL of each standard (highest to lowest), controls (low and high), or sample, were added to each well, and 50 μL of diluted Microparticle Cocktail (0.5 mL Microparticle Cocktail + 5 mL Microparticle diluent) was added, with subsequent foil plate sealing and a 2 hours incubation at room temperature on a microplate shaker. Washing involved applying a magnetic device, filling wells with Wash Buffer (100 μL), and repeating this process three times. Fifty microliters of diluted Biotin‐Antibody Cocktail (0.5 mL Biotin‐Antibody Cocktail + 5 mL Biotin‐Antibody diluent 2) was added, followed by a 1 hour incubation, and a repeat of the washing steps. Subsequently, 50 μL of diluted Streptavidin‐PE (220 μL Streptavidin‐PE – concentrated + 5.35 mL wash buffer) was added, and after a 30‐min incubation, a final round of washing was performed. Microparticles were resuspended with Wash Buffer (100 μL per well) and incubated for 2 min on the shaker. Reading was carried out within 90 min using a Luminex or Bio‐Rad analyzer, emphasizing immediate microparticle resuspension before reading by shaking the plate for 2 min at 800 ± 50 rpm. All assays were validated by the manufacturer for sensitivity, intra‐assay precision, and assay linearity. Assays were tested for less than 0.5% cross‐reactivity and interference.

The concentration of IL‐17D was quantified using the Human IL‐17D ELISA kit (Cat # EKX‐B2Y41N‐96) purchased from Nordic BioSite, following manufacturer's instructions. The concentration of hCFH was quantified using the Human Complement Factor H ELISA kit (ThermoFisher Scientific, Cat # EH122RB‐96), following manufacturer's instructions. Undiluted urine samples were used and the concentrations of IL‐17D and hCFH were determined using standard curves.

### 2.5 Immunohistochemistry

To detect specific protein responses in cells from treated patients or the placebo group, urine was collected onto cytospin slides, fixed and examined by immunohistochemistry. The cytospin slides were incubated with antibodies specific for IL‐1RA, IL‐1β, IFN‐α2, IFN‐γ, IL‐2, and TNF‐α. Chambers and cytospin slides were washed (PBS, 5 min), permeabilized (0.25% TritonX‐100 in PBS, 20 min, room temperature) and blocked (5% normal goat serum in PBS, 1 hour, room temperature) before the addition of primary antibodies (diluted in blocking buffer at 4°C overnight, or 2 hours at room temperature) including IL‐1RA (Santa Cruz, Cat # sc‐374084); IL‐1β (abcam, Cat # ab9722) TNF‐α (GeneTex, Cat # GTX110520); IL‐2 (Proteintech, Cat # 60306‐1‐Ig); Granzyme B (ThermoFisher, Cat # MA1‐80734); IFN‐γ (Invitrogen, Cat # MM700); IL‐17A (abcam, Cat # ab79056); IL‐17D (abbexa, Cat # abx129210). Slides were washed (PBS, 3 × 3 min) and stained with Alexa‐488 labeled secondary antibody (Invitrogen, Cat # A11034, 1:200, 1 h at room temperature). Nuclei were counterstained using Hoechst (1:2,000, 15 min) before a final wash (3 × 3 min in PBS). Slides were mounted (Prolong Glass Antifade Mountant, Invitrogen, Cat # P36980), before capturing images by laser scanning confocal microscopy (Carl Zeiss). Fluorescence intensity was quantified by ImageJ in three fields per sample, containing at least 50 cells per field in the treatment groups (1.7 or 8.5 mM) but fewer cells in the placebo group.

Human HTB9 bladder carcinoma cells (grade II carcinoma cells, ATCC Cat# 5637, RRID:CVCL_0126) were seeded in 6‐well flow‐chamber slides overnight (30,000 cells per well, ibidi, Cat# 80606). The cells were fixed and stained as described above.

### 2.6 RNA sequencing

RNA was extracted from tumor tissue biopsies stabilized in RNAlater, using the AllPrep DNA/RNA/miRNA Universal Kit. Disruption was performed with the TissueLyser system and CK28 Precellys tubes followed by homogenization with QIAshredder columns. The quantity and quality of RNA samples were evaluated using NanoDrop. RNA samples were prepared by Illumina TruSeq Stranded mRNA Library Prep Kit, and libraries were multiplexed and sequenced using NextSeq 500/550 High Output Kits (v2.5 2 × 75 Cycles) with an average of 22 million reads per sample. Raw sequencing data was demultiplexed using bcl2fastq (version 2.18) and RSEM (1.3) was used for abundance estimation using the human genome release 37/Ensemble 75. Samples were thoroughly quality checked and visualized using dimensionality reduction (i.e., Principal component Analysis, PCA), MA‐plots as well as RNA‐seq intrinsic biases (such as GC bias, transcriptome complexity, and alignment quality).

Differential gene expression analysis was performed using R (version 3.4) and the packages Limma and DESeq2. Fold changes were calculated by comparing tumors in the treated patients to the placebo group. Differentially expressed genes were functionally characterized using the Ingenuity Pathway Analysis version 57662101 (IPA, Qiagen) software.

### 2.7 Statistical analysis

As all data were not following a Gaussian distribution (D'Agostino & Pearson normality test) non‐parametric analysis tools were used. The Mann–Whitney *U*‐test, which is commonly used in studies of responses to BCG in urine^7^, ^17^ was selected to compare the pre‐V1 samples to the post‐inoculation samples in different groups, and to compare the treatment groups to the placebo group. The data was further analyzed using the One Way Anova, to compare the distribution of the parameters between the three patient groups. Statistical significance was determined using GraphPad (Prism v.10.0.2) and significance was assigned at *p < 0.05, **p < 0.01 and ***p < 0.001. For transcriptomic analysis, relative expression levels were analyzed and genes with an absolute fold change >1.5 and *p* < 0.05 were considered as differentially expressed. Logistic regression analysis was performed using the glm2 function of the statistics package implemented in R 4.3.1. Odds ratios were calculated from the logistic regression coefficients.

## 3 RESULTS

The response to alpha1‐oleate treatment was quantified by proteomic analysis of patient urine samples. Alpha1‐oleate was administered intravesically, on six occasions during 1‐month, preceding scheduled TURBT. Urine samples were collected longitudinally from each patient to measure urine cytokine levels before the first instillation (pre‐V1), and after each instillation of alpha1‐oleate on Days 1, 3, 5, 8, 15, and 22 (Figure S1). The response to alpha1‐oleate instillations was first evaluated by comparing pre‐instillation cytokine concentrations (Pre‐V1 samples) to post‐instillation samples. The proteomic profiles post‐instillation were subsequently compared between patients receiving 1.7 mM of alpha1‐oleate (20 patients, 120 samples) and patients receiving placebo (PBS, 20 patients, 120 samples). The dose‐dependence of the cytokine response was further analyzed by comparing post‐instillation samples in patients who received 8.5 mM of alpha1‐oleate during a dose‐finding extension of the study (6 patients, 42 samples) to samples from patients receiving 1.7 mM of alpha1‐oleate or placebo. Details about the study population demographics have been published.^6^

### 3.1 Initial proteomic analysis of urine samples

An initial screen included a bladder cancer panel with 16 proteins (MCP‐1, MIP‐1α, MIP‐1β, IP‐10, IFN‐α, IL‐1α, IL‐1RA, IL‐8, IL‐7, IL‐31, IL‐15, TNF‐β, Eotaxin, SDF‐1α, Rantes, and GRO). A significant increase in urine concentrations of IL‐1RA, IL‐1α, IL‐8, Rantes, and MIP‐1α was detected in patients treated with 1.7 mM of alpha1‐oleate, compared to placebo (Figure S2). In contrast, the concentrations of Eotaxin and IL‐7 were reduced, and the remaining cytokines in this array were not significantly affected by alpha1‐oleate treatment (Figure S2).

### 3.2 Extended proteomic analysis of urine samples from treated patients compared to the pre‐V1 samples

**FIGURE 1:**
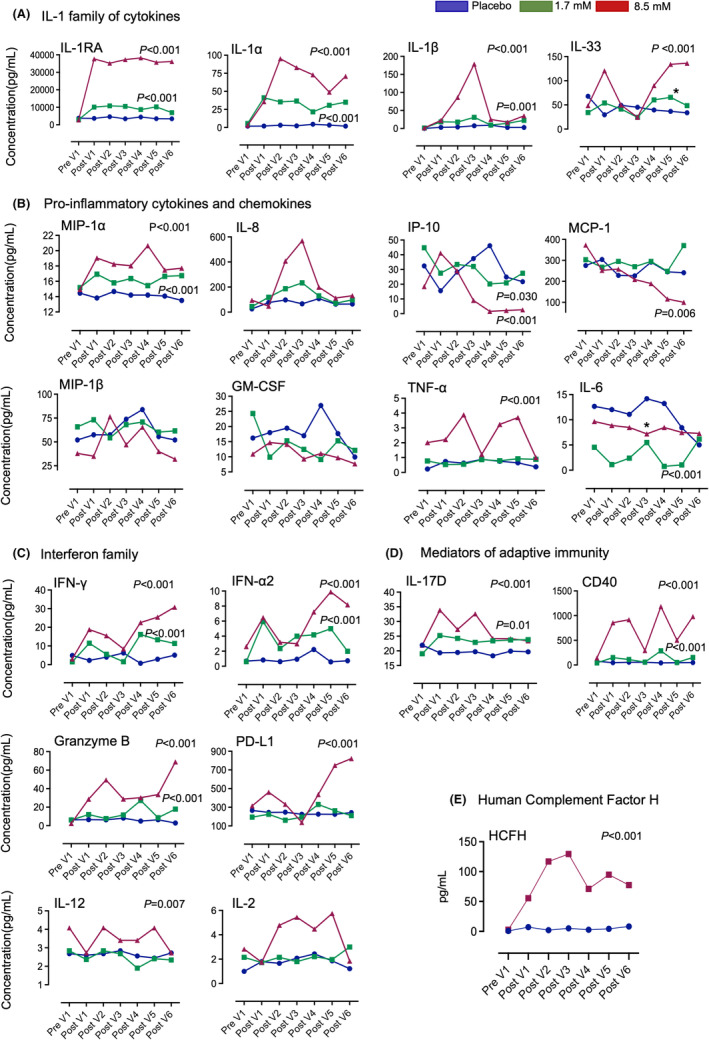
Kinetics of the cytokine response to alpha1‐oleate treatment in sequential urine samples. Patients with NMIBC were treated with 1.7 or 8.5 mM of alpha1‐oleate by intravesical instillation on six occasions (Figure S1). Cytokine concentrations were compared to a placebo group receiving the vehicle. Urine samples were analyzed for 25 cytokines. (A) The IL‐1 protein family (IL‐1RA, IL‐1α, IL‐1β, and IL‐33). IL‐1RA, IL‐1α showed a significant increase that was detected within 2 h of treatment and repeated at all visits. IL‐1β and IL‐33 showed a significant increase, with a more variable pattern. (B) The chemokine and pro‐inflammatory cytokine response. The chemokines MIP‐1α and IL‐8 were activated, while IP‐10, MIP‐1β, MCP‐1, GM‐CSF were inhibited. The proinflammatory cytokines TNF‐α increased in response to alpha1‐oleate, in contrast to IL‐6, which was reduced. (C) The interferon response. Rapid and sustained activation of IFN‐γ and IFN‐α2 was detected. (D) The adaptive immune response. IL‐17D, CD40, PD‐L1, Granzyme B, IL‐10, IL‐2, and IL‐12 showed a low initial response, followed by an increase at later visits, with different patterns. The cytokines IL‐4, IL‐13, IL‐15, and IL‐17A were not regulated (see Figure S5). (E) The hCFH showed a rapid increase in response to treatment. Mann–Whitney test comparing each of the alpha1‐oleate treated patient group (1.7 or 8.5 mM) to placebo (for analysis of this data set by One Way Anova, see Figure S4).

**FIGURE 2:**
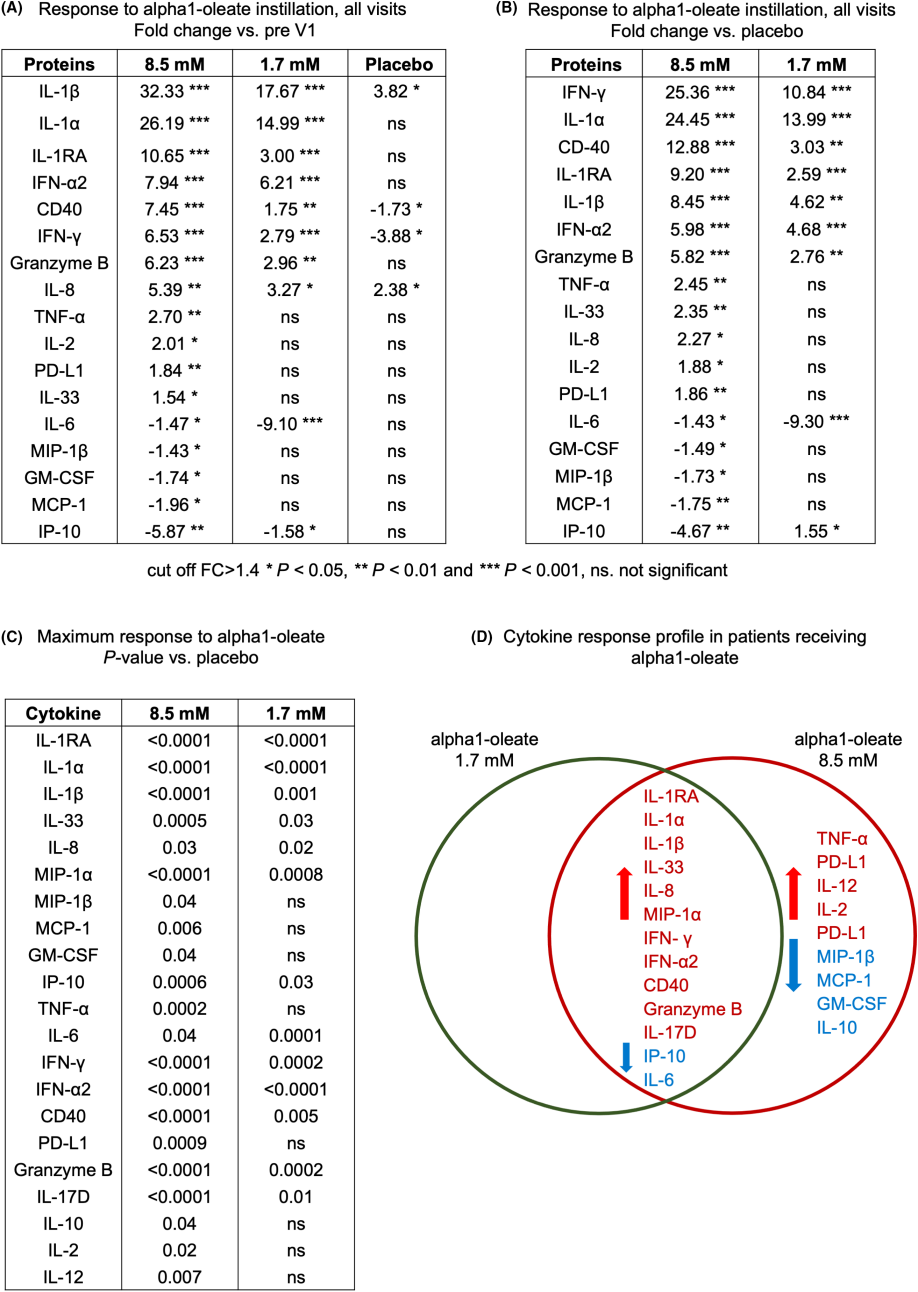
Overview of the proteomic analysis in response to alpha1‐oleate treatment. (A) Comparison of the pre‐inoculation sample obtained at Visit 1 to the post‐inoculation samples obtained a subsequent visits (fold change, median values compared to pre‐V1). Urine cytokine concentrations in the placebo group did not differ significantly from those in the pre‐V1 samples, except for IL‐1β, IL‐8, which were higher and IFN‐γ and CD40, which were lower than the urine cytokine concentrations in the pre‐V1 samples. (B) Response to alpha1‐oleate treatment defined by the fold change compared to placebo. Median values were calculated by including all samples for each cytokine and patient group. (C) Dose‐dependent increase in cytokine response, defined by the maximum protein response in each patient and treatment group, compared to placebo. (D) Venn diagram comparing the cytokine response profiles in patients receiving 1.7 or 8.5 mM of alpha1‐oleate compared to placebo. Thirteen cytokines were affected by both doses, and eight were specific for the higher dose of alpha1‐oleate (red = activated, blue = inhibited, compared to placebo). Mann–Whitney test, ***p < 0.001, **p < 0.01. *p < 0.05 (for additional analyses, see also Figure S4).

The proteomic screen was subsequently extended, to characterize the response to alpha1‐oleate in greater detail (Figures 1 and 2). Urine samples were analyzed for 24 proteins using the Luminex human immunotherapy panel (MCP‐1, MIP‐1α, MIP‐1β, CD40, IP‐10, GM‐CSF, Granzyme B, IFN‐α2, IFN‐γ, IL‐1α, IL‐1β, IL‐1RA, IL‐2, IL‐4, IL‐6, IL‐8, IL‐10, IL‐12 p70, IL‐13, IL‐15, IL‐17, IL‐33, PD‐L1, and TNF‐α). There was no significant difference in urine cytokine levels between the pre‐V1 samples and the post‐instillation samples in the placebo group, suggesting that the instillation procedure did not significantly alter urine cytokine levels (Figure S3). Comparing the pre‐V1 samples to the post instillation samples, the most rapid response, with increased cytokine levels at visit 1, was observed for IL‐1RA, IL‐1α, IL‐1β, IFN‐α2, and IL‐17D, followed by IL‐33, MIP‐1α, TNF‐α, IFN‐γ, CD40, Granzyme B, PDL‐1, IL‐2, and IL‐12 responses at subsequent visits (Figure 1).

The rapid innate immune response observed in the initial screen was confirmed in the extended screen, by comparing urine cytokine concentrations in the post‐inoculation samples in patients treated with 1.7 or 8.5 mM of alpha1‐oleate to the placebo group (Figures 1 and 2, groups compared using the Mann–Whitney test, Figure S4, groups compared using One‐Way Anova). This included the IL‐1 family of cytokines (IL‐1RA, IL‐1α, IL‐1β, and IL‐33). IL‐1RA concentrations in urine were elevated from about 3,000 pg/mL in the pre‐treatment samples to >35,000 pg/mL post instillation, and IL‐1α from 2 to >80 pg/mL. A significant increase after each instillation suggested a continuing IL‐1RA response to alpha1‐oleate during the treatment period. IL‐1α showed an initial increase from 2 to >180 pg/mL, followed by a decline after visit 4. The IL‐33 response was activated from 30 to >150 pg/mL, except at visit 3 (Figure 1A). The proinflammatory cytokines TNF‐α and the chemokine MIP‐1α showed a rapid initial increase, and concentrations in urine remained elevated during the treatment period, compared to placebo. The IL‐8 response was transiently increased at visits 2–3 (Figure 1B).

Alpha1‐oleate treatment further activated an interferon response. The median concentration of IFN‐γ increased from <5 to >30 pg/mL and IFN‐α2 from <5 to >10 pg/mL and both IFN‐α2 and IFN‐γ remained high during the treatment period (Figure 1C). In contrast, certain proteins with elevated urine levels prior to treatment showed a decline post‐treatment (MCP‐1, IP‐10, and MIP‐1β). IL‐6 concentrations were also reduced over time (Figure 1B).

Molecules associated with adaptive immunity were affected by alpha1‐oleate treatment. CD40, PD‐L1, Granzyme B, and IL‐17D were rapidly activated and concentrations in urine increased over time. IL‐2 and IL‐12 concentrations increased during the treatment period, except for a reduction at the last visit (Figure 1D). In contrast, IL‐4, IL‐13, IL‐15, and IL‐17A did not change in response to treatment, compared to the placebo group (Figure S5).

### 3.3 Dose‐dependence of the cytokine response

The dose‐dependence of the cytokine response was examined by comparing samples from patients treated with 1.7 mM of alpha1‐oleate to samples from patients receiving 8.5 mM of alpha1‐oleate (Figures 1 and 2; Figure S4). A dose‐related effect on the cytokine response was detected in patients treated with 8.5 mM of alpha1‐oleate compared to 1.7 mM of alpha1‐oleate or placebo. The concentrations of IL‐1RA, IL‐1α, IL‐1β, IL‐33, MIP‐1α, TNF‐α, IFN‐α2, IFN‐γ, CD40, PD‐L1, Granzyme B, IL‐12, and IL‐17D were significantly higher in the 8.5 mM treatment group than in the 1.7 mM treatment group. In contrast, MCP‐1, MIP‐1β, and IP‐10 concentrations were significantly lower in the 8.5 mM treatment group than in the 1.7 mM treatment group (Figure 1). Increased IL‐12, TNF‐α, IL‐2, and PD‐L1 responses were only observed in the 8.5 mM treatment group and a dose‐dependent increase in urine IL‐17D concentrations was detected, compared to the placebo group. IL‐17A concentrations were low in the groups treated with 1.7 mM or 8.5 mM of alpha1‐oleate (Figure S5).

### 3.4 Cellular origin of the cytokine response

Alpha1‐oleate instillations activate rapid tumor cell shedding into the urine, with a significant increase in cell numbers, 2 h after each instillation.^6^, ^23^ This response was observed in all patients receiving 8.5 mM of alpha1‐oleate, and in most patients receiving 1.7 mM of alpha1‐oleate. Shed cells in urine were harvested by cytospin onto microscopy slides and were stained by immunocytochemistry to examine cellular cytokine content. Cytospin samples were selected for analysis based on the proteomic data, from visits where the urine concentrations of the respective cytokine were increased. The fluorescence intensity for each cytokine was quantified and the alpha1‐oleate treated samples were compared to the pre‐instillation sample for each patient, or to the placebo group (3 patients investigated per group, 3 visits per patient in the treated group receiving 1.7 or 8.5 mM of alpha1‐oleate or the placebo group).

**FIGURE 3:**
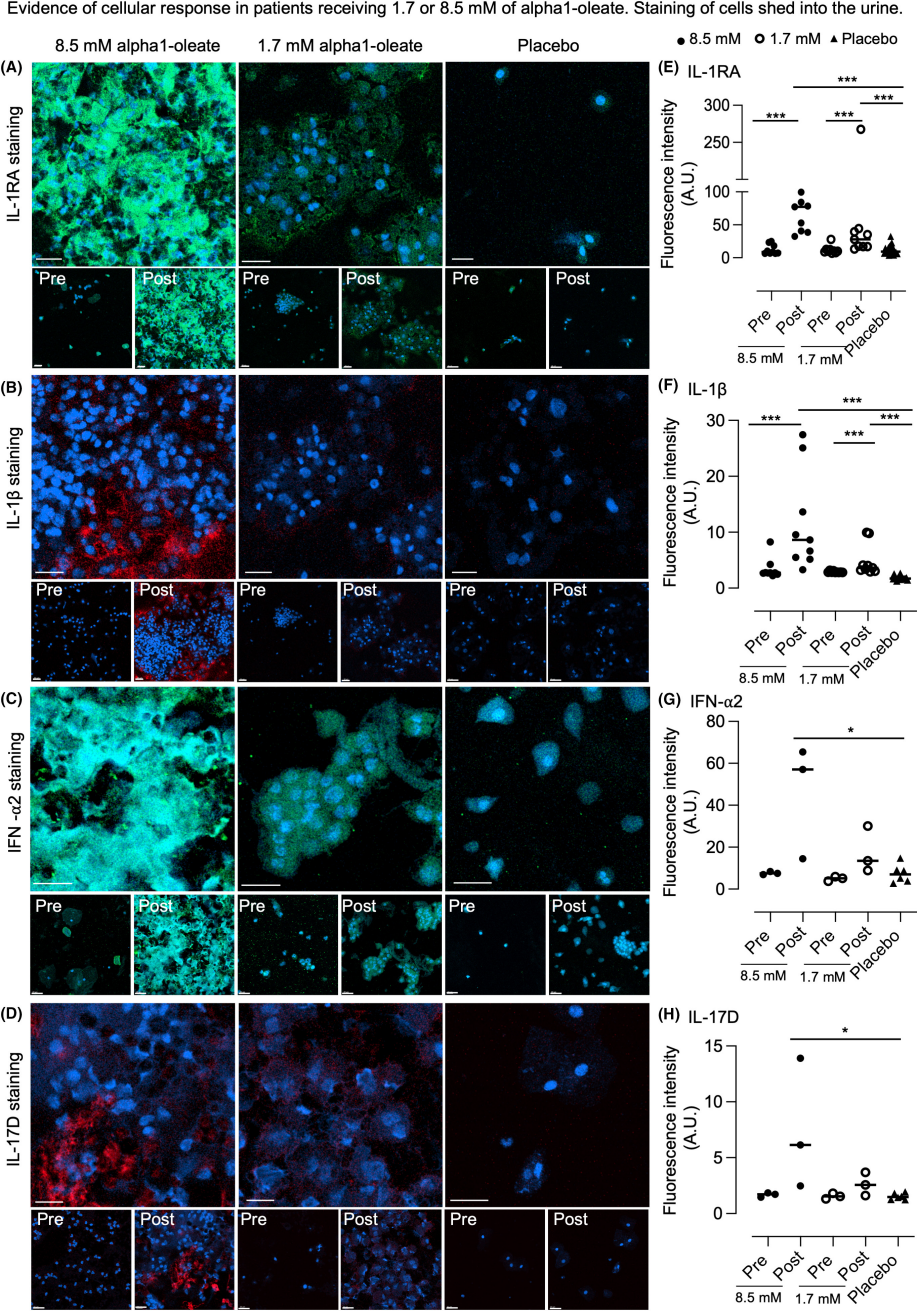
Cellular response to alpha1‐oleate, analyzed by immunocytochemistry. Cytokine staining was quantified in cells harvested from the urine of patients treated with alpha1‐oleate (1.7 or 8.5 mM) or placebo, after staining with specific antibodies. Samples obtained before instillation were compared to samples obtained within 2 hours post‐instillation. Samples from patients treated with alpha1‐oleate were compared to samples from the placebo group. (A–D) A rapid increase in cellular IL‐1RA (A), IL‐1β (B), IFN‐α2 (C), and IL‐17D (D) content was observed after instillation of alpha1‐oleate, compared to the pre‐instillation sample. (E‐H) Quantifications of staining in (A–D), quantified by imageJ. Three fields imaged per sample. IL‐1RA, IFN‐α2 are represented in green, IL‐1β, IL‐17D are represented in red, nuclei stained with hoechst in blue. The cellular response was significantly higher in the treatment groups than in the placebo group, and higher in the 8.5 mM treated group than in the 1.7 mM group. In contrast, there was no increase in staining for IL‐2, Granzyme B, IFN‐γ or TNF‐α and responses were low (Figure S13). *N* = 3–9, Mann–Whitney test, ***p < 0.001, **p < 0.01. *p < 0.05. Scale bar 30 μm.

A significant increase in cellular staining was observed for IL‐1RA, IL‐1β, IFN‐α2, and IL‐17D compared to pre‐V1 samples in each treated group, and to the placebo group (Figure 3). Furthermore, the IL‐1RA cellular response was significantly higher in the 8.5 mM treated group than in the 1.7 mM group, consistent with the dose‐dependent increase in urine protein concentrations (Figure 3A; Figure S7). A similar increase in cellular staining was observed for IL‐1β, compared to the pre‐instillation samples in each patient, and to samples from the placebo group. Cellular IL‐1β staining was significantly higher in the 8.5 mM treated group than in the 1.7 mM group, corresponding to the urine proteomic data (Figure 3B; Figure S8). Furthermore, increased cellular staining for IFN‐α2 and IL‐17D was detected in shed cells (3 patients investigated, 1 visit per patient, Figure 3C,D; Figure S9). The results identify a strong and rapid local response to alpha1‐oleate instillation, in cells shed from the urinary bladder (Figure 3E–H).

While the cellular origin of the shed cells is not possible to determine, morphologic analysis defined the majority of cells as epithelioid, suggesting an origin from the bladder mucosa and the tumor. Cells with lymphoid morphology were not abundant among the shed cells and gene expression analysis detected inhibition rather than activation of immune response genes in tumor tissue.

To address if bladder cancer cells do produce some of the key cytokines detected in the treated patients, human bladder carcinoma cells (HTB‐9) were investigated by immuno‐histochemistry, using specific antibodies (Figure S10). Significant levels of IL‐1RA, IL‐1β, TNF‐α, IFN‐γ, IL‐2, IL‐17A, IL‐17D, and Granzyme B were detected, with a pattern of strong nuclear staining for IL‐1RA, IL‐1β, Granzyme B and IL‐17A, compared to the cytoplasmic staining.

### 3.5 Response to alpha1‐oleate treatment defined by RNA sequencing of tumor tissue

RNA isolated from tissue biopsies, collected post treatment at TURBT, was subjected to RNA sequencing. Changes in gene expression in response to alpha1‐oleate treatment were quantified by comparing tissue samples from the alpha1‐oleate treated groups (1.7 or 8.5 mM) to tissues obtained from the placebo group.

**FIGURE 4:**
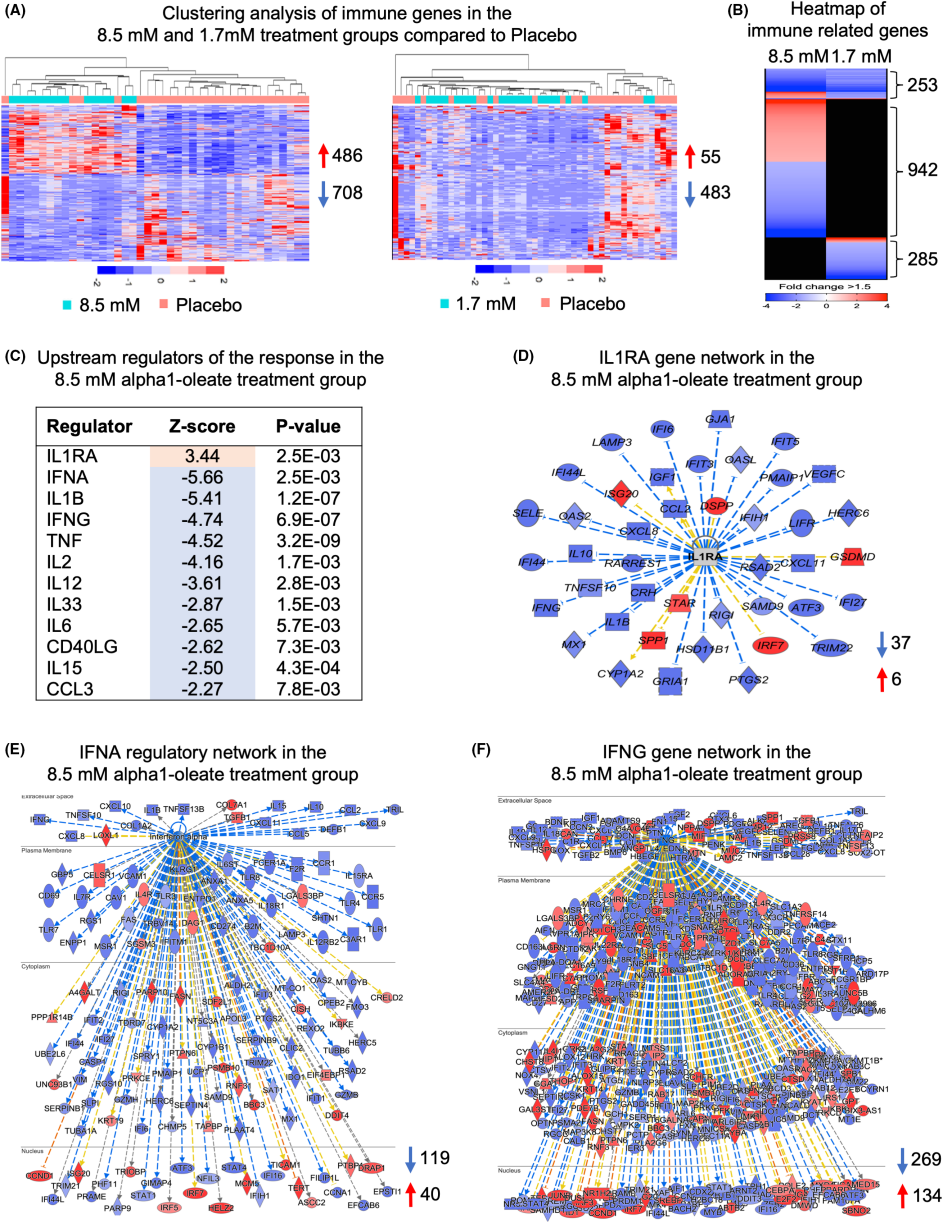
Gene expression profiling in tumor tissues from patients receiving 1.7 or 8.5 mM of alpha1‐oleate compared to placebo. (A) Heatmap clustering based on differentially expressed immune related genes between tumors in the 1.7 or 8.5 mM treatment groups and the placebo group (cutoff FC >1.5, *p* < 0.05, compared to placebo, red = upregulated, blue = downregulated). (B) Heatmap comparison between 1.7 and 8.5 mM treated tumors. (C) Upstream regulator analysis of 8.5 mM treated tumors predicting *IL1RA* to be activated and 11 immune related genes including *IFNA* and *IFNG*, *IL1B* and *TNF* to be strongly inhibited (z‐score ≤−2, *p* < 0.05). (D) *IL1RA* gene network in 8.5 mM treated tumors. (E) *IFNA* gene network showing strong inhibition in 8.5 mM treated tumors. (F) *IFNG* gene network showing strong inhibition in 8.5 mM treated tumors.

Expression profiling detected significant changes in tumors from patients treated with 1.7 or 8.5 mM of alpha1‐oleate compared to the placebo group, with a predominance of downregulated genes in both groups (Figure 4A). The effect was significantly higher in the 8.5 mM treatment group than in the 1.7 mM treatment group consistent with the increased antitumor effect. In addition to the overall downregulation of genes, the immune‐related genes were also downregulated in both treatment groups compared to placebo (Figure 4A), with a stronger effect in the 8.5 mM compared to 1.7 mM treatment group (708 and 483 downregulated genes, respectively). A total of 253 immune‐related genes were shared between the two treatment groups and the majority of these genes showed reduced expression compared to placebo (Figure 4B).

Next, the effect on the immune‐related genes was functionally categorized using IPA, which provides insights into biological pathways, molecular networks, and functional annotations related to gene expression data, after extensive database and literature search. Gene expression data showed strong regulation of immune related functions in both 1.7 and 8.5 mM treatment groups (Figure S11A,B). According to the IPA analysis, innate immune response biofunctions were strongly inhibited in both 1.7 and 8.5 mM groups, suggesting an acute anti‐inflammatory response to alpha1‐oleate treatment. Cytokine storm genes, which include genes related to phagocytes, monocytes, granulocytes, and neutrophils were strongly downregulated in both treatment groups with a higher decrease in the 8.5 mM treated group. Furthermore, genes related to adaptive immune response functions were shown to decrease with higher treatment dose.

The inhibition of gene expression was attributed, in part, to IL‐1RA and its inhibitory effects on innate immunity. *IL1RA* was identified as the top upstream regulator of the immune response to alpha1‐oleate in the 8.5 mM group (Figure 4C,D), potentially explaining the inhibition of innate immune related genes. Furthermore, *IFNA* and *IFNG* were both identified as major upstream regulators of the cytokine response, including adaptive immunity (Figure 4E,F).

**FIGURE 5:**
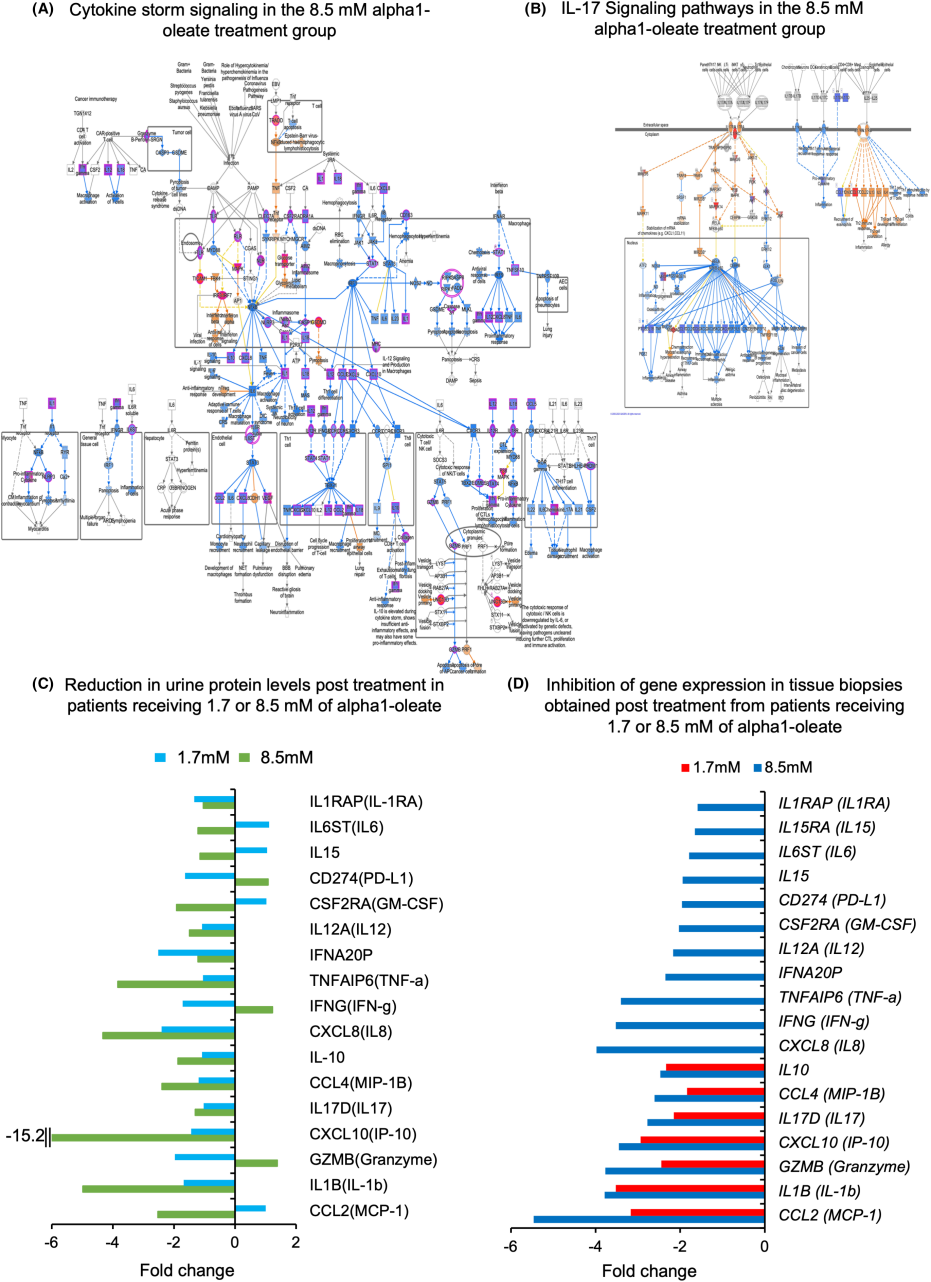
(A, B) Functional analysis of gene expression data identifying pathways that were regulated in tumor tissues from patients receiving 8.5 mM of alpha1‐oleate, compared to placebo. (A) Strong downregulation of the vast majority of cytokine storm signaling pathway genes in the 8.5 mM treated tumors is shown, compared to the placebo group, consistent with the strong inhibition of innate immunity. (B) Downregulation of *IL17D* and genes in the IL‐17 signaling pathway in the 8.5 mM treated tumors compared to placebo, and parallel activation of the IL‐17A inhibitor *IL17RC* is shown (red = upregulated, blue = downregulated, cut off fold change 1.5, *p* < 0.05). (C, D) Comparison of gene expression and urine proteomic analysis at the end of alpha1‐oleate treatment. (C) Several proteins, showed an initial increase in urine protein concentrations, followed by a decrease at the last visit (fold change <−1, see also Figure 1). Cytokines with this response pattern included MCP‐1, IL‐1β, IP‐10, TNF‐α, MIP‐1β, IL‐10, GM‐CSF, IL‐6, IL‐8, and IL‐17, which showed low levels in urine at the time of surgery, when the tissue biopsies for RNA analysis were obtained. (D) RNA sequencing revealed a corresponding pattern of downregulation, showing that the expression of the corresponding genes was inhibited in treated tumor tissue, compared to placebo group.

Canonical pathway analysis further identified cytokine storm signaling as strongly inhibited by alpha1‐oleate (Figure 5A; Figure S11E), broadly affecting cytokines and their receptors and predicting a general inhibition of inflammation and innate immunity. The IL‐17 signaling pathway was inhibited in both treatment groups compared to placebo, including *IL17D*, CCL2 (*MCP1*), *IL1B* and Defensin beta 1 (*DEFB1*) (Figure 5B; Figure S11F). Downregulated genes in the IL‐17 pathway also included growth factor receptors, ion channels and Ras signaling, as well as and regulators of gene expression.

### 3.6 Long‐term proteomic and RNA response to alpha1‐oleate

The sequential intravesical inoculations and longitudinal collection of urine samples made it possible to evaluate the response pattern over time by comparing later samples to the early response. Furthermore, the change over time from the acute response to the end of the treatment period could be evaluated in individual patients. Two different patterns were observed in patients receiving 8.5 mM of alpha1‐oleate. First, elevated cytokine concentrations throughout the treatment period were detected for IL‐1RA and the IFN‐α2 and IFN‐γ, with responses increasing with the treatment time. Second, an initial increase followed by a reduction in cytokine concentrations toward the end of the treatment period was detected for several cytokines, suggesting that immune reactivity might be reduced by treatment (Figure 5C). This reduction was quantified by comparing the highest concentration of each cytokine recorded during treatment to the last sample obtained after the instillation at visit 6 (MCP‐1, IL‐1β, IP‐10, IL‐17D, MIP‐1β, IL‐10, IL‐8, TNF‐α, IL‐12, GM‐CSF, IL‐15, and IL‐6) (Figure 5C). A response characterized by initial activation followed by a reduction was also detected for the bladder cancer biomarker hCFH, which was quantified by ELISA in urine samples from the 8.5 mM alpha1‐oleate treatment group compared to the placebo group (Figure 1E).

The gene expression data from tumor tissue were subsequently compared to the urine proteome at the last visit (V6). A reduction of urine protein concentrations was observed for the specific genes that were regulated at the RNA level, with more potent inhibition in the 8.5 mM than in the 1.7 mM treatment group (Figure 5D).

### 3.7 Cytokine response and treatment outcome

**FIGURE 6:**
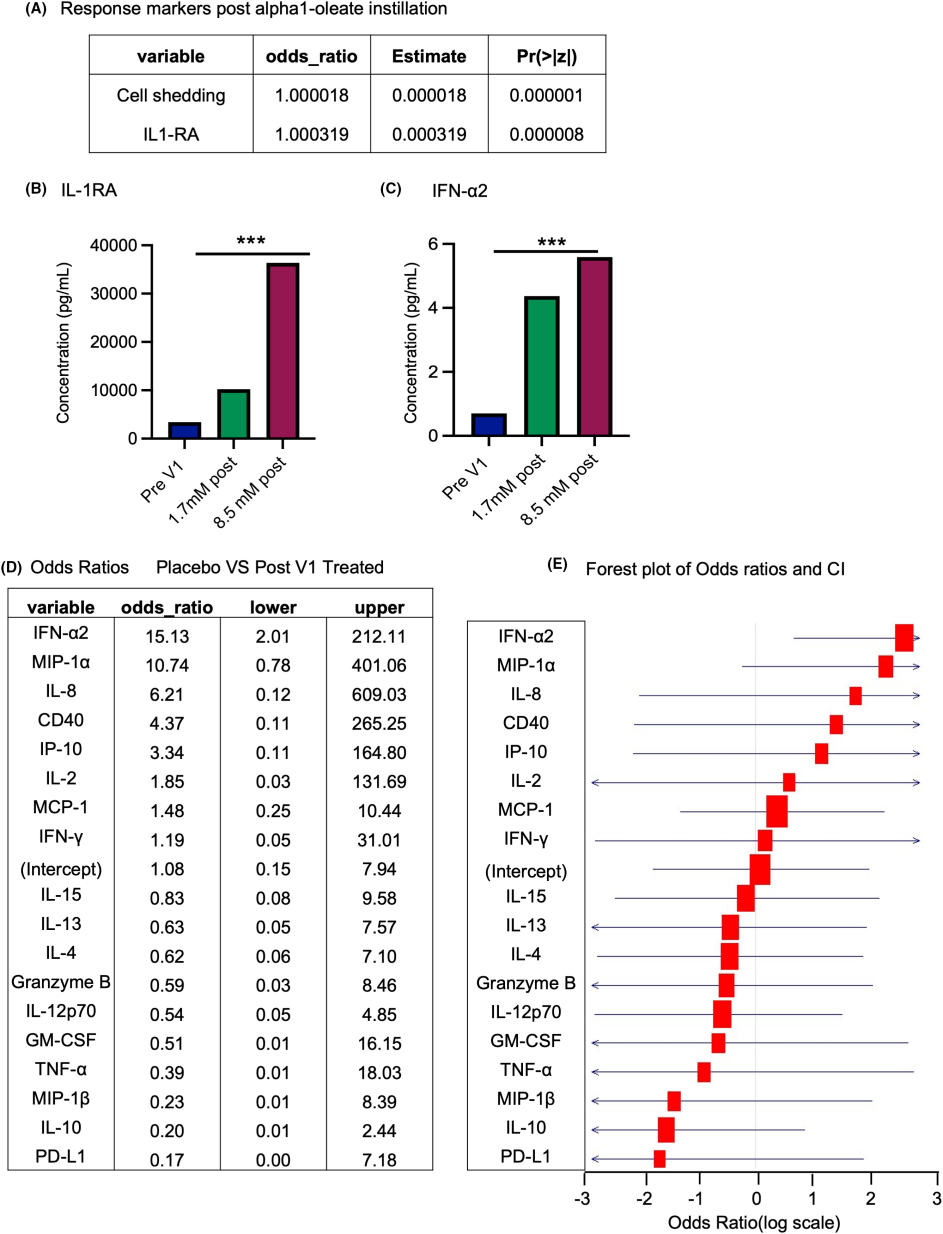
Significant association between alpha1‐oleate treatment, IL‐1RA and the likelihood of cell shedding. (A) Logistic regression analysis of IL‐1RA protein levels and tumor cell shedding in relation to alpha1‐oleate exposure. The analysis was performed using *glm* package in R, revealing significant association between alpha1‐oleate treatment and the likelihood of IL‐1RA and cell shedding. Odds ratios and confidence intervals were calculated to quantify the strength of the relationships. (B, C) IL‐1RA and IFN‐α2 protein concentrations in samples obtained before treatment (Pre‐V1) or post treatment (V1–V6) in patients treated with 1.7 or 8.5 mM of alpha1‐oleate. (D, E) Odds ratios and confidence intervals for protein levels predicted against alpha1‐oleate treatment. IFN‐α2, MIP‐1α, IL‐8, and CD40 showed an odds ratio of >2 suggesting concentration of these proteins is more likely to be higher with alpha1‐oleate treatment compared to placebo. The red color represents the log odd ratio, and the horizontal line represents confidence intervals of each protein.

To examine if the cytokines serve as predictors of treatment effects, Logistic Regression Analysis of protein levels and tumor cell shedding was studied, using the *glm* package in R. A significant association between alpha1‐oleate treatment, cell shedding, and the IL‐1RA response was detected. The results showed a significant association of alpha1‐oleate treatment with increased cell shedding (odds ratio >1 and *p* < 0.0001), and the likelihood of IL‐1RA activation, suggesting IL‐1RA as a predictor of cell shedding and a response marker of alpha1‐oleate treatment (Figure 6A,B).

Similarly, odds ratios were estimated to identify cytokines that predict the likelihood of an immune response to alpha1‐oleate treatment. Interestingly, an increase in IFN‐α2 protein concentrations predicted outcome defined by the remaining cytokines in the alpha1‐oleate treated group with an odd ratio >15, suggesting IFN‐α2 as a potential response marker of the immune response to alpha1‐oleate treatment (Figure 6C–E).

The effect on gene expression suggested that alpha1‐oleate treatment triggers an acute increase in cytokine levels, which is maintained throughout treatment for IL‐1RA and IFN‐α2, which also predict cell shedding and innate immune activation. For a subset of cytokines, the acute response is followed by a later reduction, possibly reflecting the tumor response to treatment and removal of tumor cells from the tissues.

### 3.8 Comparison of the urine proteomes in alpha1‐oleate treated and BCG treated patients

**FIGURE 7:**
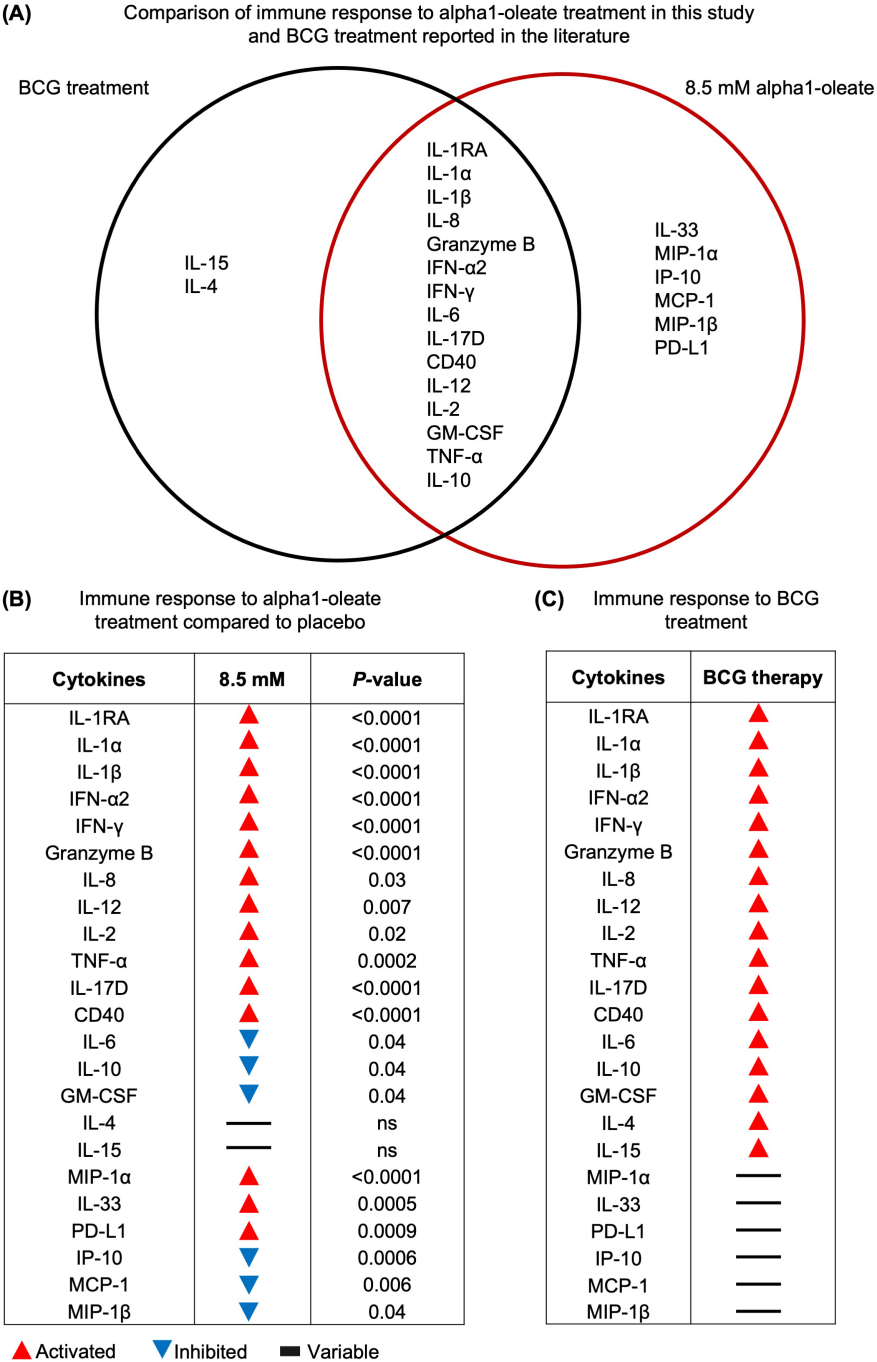
Comparative analysis of the response to alpha1‐oleate in this study and the response to BCG treatment, reported in the literature. (A) Venn diagram visualizing the overlap in cytokine response profiles between patients treated with alpha1‐oleate (8.5 mM) in this study and BCG treated patients, reported in the literature (*p* < 0.05). (B, C) Comparison of cytokine responses detected in alpha1‐oleate treated patients and responses to BCG treatment. *p* values are shown for the 8.5 mM alpha1‐oleate treatment group versus placebo. Arrows indicate activation (red) or inhibition (blue), variable indicates non‐conclusive literature data (black). References for response to BCG treatment: IL‐1family,^13^, ^16^, ^17^, ^18^, ^22^, ^25^, ^29^, ^30^, ^32^ IFN‐α2,^16^, ^17^, ^18^, ^19^, ^22^, ^24^, ^29^, ^30^, ^33^, ^34^ IFN‐γ,^16^, ^17^, ^18^, ^21^, ^22^, ^27^, ^33^, ^34^, ^35^ Granzyme B,^17^, ^18^, ^29^, ^30^ IL‐8,^15^, ^16^, ^17^, ^18^, ^21^, ^22^, ^24^, ^29^, ^30^ IL‐12,^16^, ^17^, ^18^, ^21^, ^22^, ^24^, ^29^, ^30^ IL‐2,^13^, ^15^, ^16^, ^17^, ^18^, ^21^, ^22^, ^24^, ^27^, ^29^, ^30^ TNF‐α,^13^, ^16^, ^17^, ^18^, ^21^, ^27^, ^29^, ^30^, ^36^ IL‐6,^13^, ^15^, ^16^, ^17^, ^18^, ^21^, ^24^, ^25^, ^29^, ^30^, ^37^, ^38^, ^39^ IL‐10,^17^, ^18^, ^24^, ^26^, ^29^, ^30^ GM‐CSF,^17^, ^18^, ^21^, ^22^, ^24^, ^25^, ^27^, ^29^, ^30^ IL‐4,^16^, ^17^, ^18^, ^22^, ^24^, ^29^, ^30^ CD40,^16^, ^17^, ^40^, ^41^, ^42^ IL‐17,^16^, ^17^, ^18^, ^21^ IL‐15.^17^, ^18^, ^24^, ^29^, ^30^

The response to alpha1‐oleate treatment was subsequently compared to the immune response to BCG, reported in the literature.^15^, ^20^, ^24^, ^25^, ^26^, ^27^, ^28^, ^29^, ^30^, ^31^ A significant overlap between the two treatments is illustrated by the Venn diagrams in Figure 7A; Figure S12. Several proteins reported to increase during BCG treatment were activated in patients treated with alpha1‐oleate as well. These included IL‐1RA, IL‐1α, IL‐1β, IL‐8, IFN‐α2, IFN‐γ, and Granzyme B, which were activated in the 1.7 and the 8.5 mM treatment groups (Figure 7B,C; Figure S12). The most rapid response to alpha1‐oleate was observed for IL‐1RA, IL‐1α, IL‐1β, MIP‐1α, TNF‐α, IFN‐α2, and IFN‐γ, at the first visit. This response was more rapid than that reported in BCG treated patients.^17^ The maximum response to alpha1‐oleate was detected for IL‐1RA, with an increase to about 35,000 pg/mL compared to around 2,000–3,000 pg/mL in BCG treated patients.^17^ The comparisons with the literature further suggested that the IL‐1β, IFN‐α2, and IFN‐γ responses to alpha1‐oleate were higher than those reported for BCG treated patients.^12^, ^16^, ^17^, ^21^, ^24^

Additional proteins, that have been reported to increase during BCG treatment, were activated in patients receiving 8.5 mM of alpha1‐oleate, including IL‐2, IL‐12, TNF‐α, and IL‐17D. In contrast IL‐6 and GM‐CSF, which are activated by BCG treatment, were inhibited in alpha1‐oleate treated patients in the 1.7 mM and the 8.5 mM treatment groups. Increased levels of IL‐33, MIP‐1α, and CD40, and decreased levels of IP‐10 were observed in the alpha1‐oleate treated groups but have not been reported in BCG treated patients, suggesting additional effects of alpha1‐oleate (Figure 7B,C). IL‐4 and IL‐15 responses, which occur in BCG treated patients, were not observed in the alpha1‐oleate treatment groups.

## 4 DISCUSSION

BCG immunotherapy is the preferred treatment for high‐risk NMIBC and an option for intermediate‐risk NMIBC.^21^ The protective effect of BCG is attributed to the immune response, triggered by intravesical instillations of the live, attenuated *Mycobacterium bovis* strain in the BCG preparation. The profile of cytokines activated by BCG therapy has been extensively characterized, and several cytokines have been directly associated with the protective effects of BCG immunotherapy. In this study, a significant overlap in cytokine response profiles was demonstrated between BCG treated patients, reported in the literature, and alpha1‐oleate treated patients studied here. This included IL‐1RA, IL‐1α, IL‐1β, IFN‐α2, IFN‐γ, Granzyme B, IL‐8, IL‐12, IL‐2, and TNF‐α, which were elevated in urine samples from treated patients. In contrast, IL‐6 and GM‐CSF were inhibited and IL‐4 and IL‐15 were unaffected by alpha1‐oleate treatment. The results suggest that alpha1‐oleate and BCG immunotherapy trigger similar local host response profiles in patients with NMIBC, including cytokines proposed to contribute to the antitumor effects of BCG in this patient group.^21^, ^24^, ^25^, ^26^, ^27^, ^28^, ^29^, ^30^

The innate and adaptive immune systems are activated in patients receiving BCG immunotherapy and both have been associated with protection against bladder cancer. BCG bacteria activate pathogen‐associated molecular pattern (PAMP) signaling through Toll‐like receptors and the downstream innate immune response includes pro‐inflammatory cytokines, such as the IL‐1 family, TNF and chemokines such as IL‐8 and GM‐CSF, which promote the recruitment and activation of neutrophils and mononuclear cells in the bladder wall.^24^ BCG also stimulates a TH1 response by activating CD4^+^ T cells, which are considered essential for BCG‐mediated effects on bladder cancer.^25^, ^31^ Inducing IFN‐γ and blocking IL‐10 further stimulates the TH1‐dominated immune response, a pattern also seen in alpha1‐oleate treated patients.^35^, ^43^ While the cytokine response profile in alpha1‐oleate treated patients resembled that reported in BCG treated patients, there were differences in kinetics and magnitude. Alpha1‐oleate triggered a more rapid response than BCG, with significantly higher cytokine levels than those reported in BCG treated patients. The IL‐1RA, IL‐1β and interferon responses to BCG were detected later than in patients treated with alpha1‐oleate, as were Granzyme B, TNF‐α, IL‐2, GM‐CSF, IL‐10, IL‐12, IL‐15, and IL‐17.^17^ The results suggest that alpha1‐oleate therapy may reproduce important aspects of BCG therapy and that combination therapy might be beneficial to maximize the effect on bladder cancer progression.

The rapid innate immune response in alpha1‐oleate treated patients included molecules previously associated with improved outcome of bladder cancer, such as IL‐1RA, which was strongly activated and remained high throughout the treatment period. IL‐1RA was identified as the top upstream regulator of the innate immune response in the entire data set. The IL‐1 response to alpha1‐oleate also included IL‐1α, IL‐1β, and IL‐33, with strong pro‐inflammatory effects. IL‐1RA and IL‐1β were detected in shed tumor cells from the treated patients, as well as IFN‐α2 and IL‐17D. The cellular origin of the IL‐1 response in BCG treated patients has not been defined, but *Escherichia coli* infections are known to trigger rapid IL‐1 responses the urinary bladder mucosa, where they may drive disease progression and pain, especially in acute cystitis and patients susceptible to bladder pain syndrome.^44^, ^45^ The IL‐1 receptor antagonist IL‐1RA is a registered drug, which broadly affects the diverse clinical effects of IL‐1 hyper‐activation and IL‐1RA treatment has been shown to attenuate the symptoms in patients with bladder pain syndrome.^46^ In this study, a strong IL‐1RA response was identified in cells that were shed into the urine in response to alpha1‐oleate, confirming the local tissue response to alpha1‐oleate treatment. Based on previous reports associating elevated IL‐1RA protein levels with a reduced risk of recurrence in bladder cancer,^17^, ^18^ it may be speculated that the strong IL‐1RA response might contribute to the effect of the alpha1‐oleate complex on treated tumors.

IFN‐α2 and IFN‐γ were activated by alpha1‐oleate, as shown by elevated protein levels in urine throughout the treatment period. Anticancer properties of IFN‐α2 include direct antiproliferative effects and complex immunomodulatory effects, which are being explored for bladder cancer treatment,^33^ including adenovirus vector delivery technology.^47^ IFN‐α induces TNF‐Related Apoptosis‐Inducing Ligand (TRAIL) expression in bladder cancer cells^36^, ^47^ and triggers apoptosis in cells expressing the appropriate cell death receptor, by Fas‐associated death‐domain‐ (FADD‐) dependent activation of caspase‐8.^19^, ^34^ IFN‐γ is cytotoxic and initiates apoptosis in tumor cells together with Granzyme B and perforin.^48^, ^49^ IFN‐α and IFN‐γ have also been shown to inhibit tumor angiogenesis.^50^ Other direct effects of IFN‐α include cytotoxicity and upregulation of MHC class I expression^51^, ^52^ and IFN‐γ stimulates the synthesis of immune checkpoint inhibitory molecules and indoleamine‐2,3‐dioxygenase (IDO), thus stimulating other immuno‐suppressive mechanisms.^53^ As alpha1‐oleate triggers apoptosis in bladder cancer tissues, inhibits VEGF expression and enhances cytokine secretion, it may be speculated that the properties of alpha1‐oleate may enhance the antitumor effects of IFN‐α2 and IFN‐γ. In addition to high IFN‐γ responses and the reduced IL‐10 levels in alpha1‐oleate treated patients may potentially support a TH1 response,^4^, ^43^, ^48^ as seen also in BCG treated patients.

IL‐17D was regulated by alpha1‐oleate treatment at the protein level, suggesting that the IL‐17 network might play an immunomodulatory role in alpha1‐oleate treated patients. The regulation of IL‐17 may also provide a molecular path for the translation of innate to adaptive immunity. Adaptive immune response markers affected by alpha1‐oleate treatment, included CD40, which is a member of the TNF receptor superfamily, present on the surface of antigen‐presenting cells, such as B‐cells, macrophages/monocytes, and dendritic cells.^40^, ^54^ CD40‐mediated signal transduction stimulates the synthesis of TNF‐α, IL‐1, IL‐6, and IL‐8 by peripheral blood mononuclear cells.^41^ Moreover, CD40 may induce the production of IFN‐γ, TNF‐α, and IL‐2, consistent with the response profile in alpha1‐oleate treated patients.^42^ Anti‐TNF therapy of autoimmune disorders has been associated with an increased risk for malignancies including bladder cancer.^55^ Blocking of IL‐6 signaling has been discussed as a potential therapeutic strategy for cancers characterized by pathological IL‐6 overproduction.^37^ IL‐6 has also been shown to enhance endothelial cell migration, a key step in angiogenesis, and dissemination of solid tumors.^38^, ^39^ The reduction in IL‐6 levels by alpha1‐oleate may therefore contribute to its antitumor effects.

The results suggest that in addition to killing tumor cells, alpha1‐oleate activates a strong antitumor response that is not present in the placebo group. The predominance of IL‐1RA and interferons support the potential of the innate response as an antitumor defense. In addition, the later adaptive immune response, dominated by CD40 and PD‐L1, suggests active reprogramming of the tumor environment and evolution of adaptive immunity, possibly supported by the IL‐17 response and Granzyme B to mediate apoptosis. The eventual reduction in most of the cytokines may reflect the loss of tumor tissue and therefore tumor reactivity to alpha1‐oleate. The peptide‐based alpha1‐oleate complex is currently being investigated in patients with NMIBC, who are not eligible for BCG therapy, due to early, less severe disease. Efficacy has been demonstrated in a placebo‐controlled study using the reduction in tumor size and cell shedding as end points,^6^ suggesting that alpha1‐oleate may be useful in this patient group, where non‐toxic therapeutic alternatives are lacking. The similarities to patients with BCG immunotherapy may suggest a combined or sequential treatment approach, with priming and debulking of the tumor using alpha1‐oleate and subsequent BCG treatment, in patients with disease progression. The potential benefits of such approaches would require validation in future clinical trials.

The investigational drug, alpha1‐oleate, has the potential to be an effective neoadjuvant therapy for NMIBC. There is limited toxicity associated with the administration of alpha1‐oleate as nonclinical studies of alpha1‐oleate found no evidence of symptoms, or signs of effect on body weight, organ weights, gross pathology or histopathology.^5^ The ongoing clinical trial that is evaluating the safety and efficacy of alpha1‐oleate found that alpha1‐oleate is safe and well‐tolerated in this patient population and preliminary clinical data from this trial, suggests that alpha1‐oleate instillations result in dose‐dependent effects on both tumor size and cell shedding.^6^ The neoadjuvant therapy represents a novel approach in the treatment of NMIBC, which might reduce the extent of surgery, improve the efficacy of surgical removal and enhance the response of tumors to subsequent treatment. The neoadjuvant therapy may also improve treatment outcomes, such as recurrencies, by targeting subclinical cancer lesions and limiting spread within mucosa, potentially improving outcomes and quality of life for patients with this condition.

## AUTHOR CONTRIBUTIONS

**Shahram Ahmadi:** Conceptualization (equal); data curation (equal); formal analysis (equal); writing – original draft (equal); writing – review and editing (equal). **Ines Ambite:** Formal analysis (equal); methodology (equal); writing – review and editing (equal). **Antonín Brisuda:** Data curation (equal); investigation (equal). **Jaromír Háček:** Data curation (equal); investigation (equal). **Farhan Haq:** Formal analysis (equal); methodology (equal); writing – original draft (equal). **Samudra Sabari:** Data curation (equal); formal analysis (equal). **Kamala Vanarsa:** Data curation (equal). **Chandra Mohan:** Data curation (equal); supervision (equal). **Marek Babjuk:** Conceptualization (equal); supervision (equal). **Catharina Svanborg:** Conceptualization (equal); formal analysis (equal); methodology (equal); supervision (equal); writing – original draft (equal); writing – review and editing (equal).

## FUNDING INFORMATION

This study was supported by the Swedish Cancer Society (Cancerfonden), the Swedish Research Council (Vetenskapsrådet), the Royal Physiographic Society in Lund, HAMLET BioPharma and the European Union's Horizon 2020 research and innovation program under grant agreement No. 954360.

## CONFLICT OF INTEREST STATEMENT

C.S. and I.A. hold shares in HAMLET BioPharma, as a representative of scientists in the HAMLET group. Patents have been filed to protect the use of alpha1‐oleate in cancer therapy, with the scientists as inventors.

## ETHICS STATEMENT

The study was approved by the State Institute for Drug Control (SUKL) in the Czech Republic; number 273799/17‐I and the Ethics Committee of the Motol University Hospital; number EK‐786/17 (ClinicalTrials.gov Identifier: NCT03560479).

## Supporting information


Data S1.

## Data Availability

The microarray data has been deposited in the NCBI's Gene Expression Omnibus repository under accession number GSE209883. All other data that support the findings of this study are available from the corresponding author upon reasonable request.
