# Supplementary material for: Similar immune responses to alpha1‐oleate and Bacillus Calmette–Guérin treatment in patients with bladder cancer
Source: Cancer Med. 2024 Mar 29;13(7):e7091. doi: 10.1002/cam4.7091 (PMC10980842; doi:10.1002/cam4.7091)
Supplement: Supplementary file 1 — Data S1. [file CAM4-13-e7091-s001.pdf]

Supplementary material

Similar immune responses to alpha1-oleate and Bacillus Calmette-Guérin (BCG) treatment in patients with bladder cancer

Shahram Ahmadi[1], Ines Ambite[1], Antonin Brisuda[2], Jaromir Hacek[3], Farhan Haq[1], Samudra Sabari[1], Kamala Vanarsa[4], Chandra Mohan[4], Marek Babjuk[2] and Catharina Svanborg[1]*

Supplementary Fig. 1

Schedule for intravesical instillations (Days)

	0	1	3	5	8	15	22	TURB Biopsies/ RNA
**Placebo group**								
Urine sample, pre		20	20	20	20	20	20	20
Urine sample, post		20	20	20	20	20	20	
**Alpha1-oleate 1.7 mM**								
Urine sample, pre		20	20	20	20	20	20	20
Urine sample, post		20	20	20	20	20	20	
**Alpha1-oleate 8.5 mM**								
Urine sample, pre		6	6	6	6	6	6	14
Urine sample, post		6	6	6	6	6	5	

Supplementary Figure 1. Collection of urine samples and tissue biopsies

Urine samples were collected before and about 2 hours after each instillation of alpha1-oleate or placebo, on days 1, 3, 5, 8, 15 and 22 (6 visits). A total of 140 urine samples were analyzed from the 20 patients, who received 1.7 mM of alpha1-oletae, 140 who received placebo and 42 samples from the six patients, who received the higher dose of 8.5 mM of alpha1-oleate. Whole urine was used for cytokine quantification. Uncentrifuged urine samples were used for the quantification of cell shedding by microscopy and cytospin samples were used for immunohistochemistry and staining for specific cytokines to quantify the alpha1-oleate content in the shed tumor cells. Tissue biopsies, collected at TURBT at the end of treatment, were subjected to RNA sequencing.

Supplementary Fig. 2

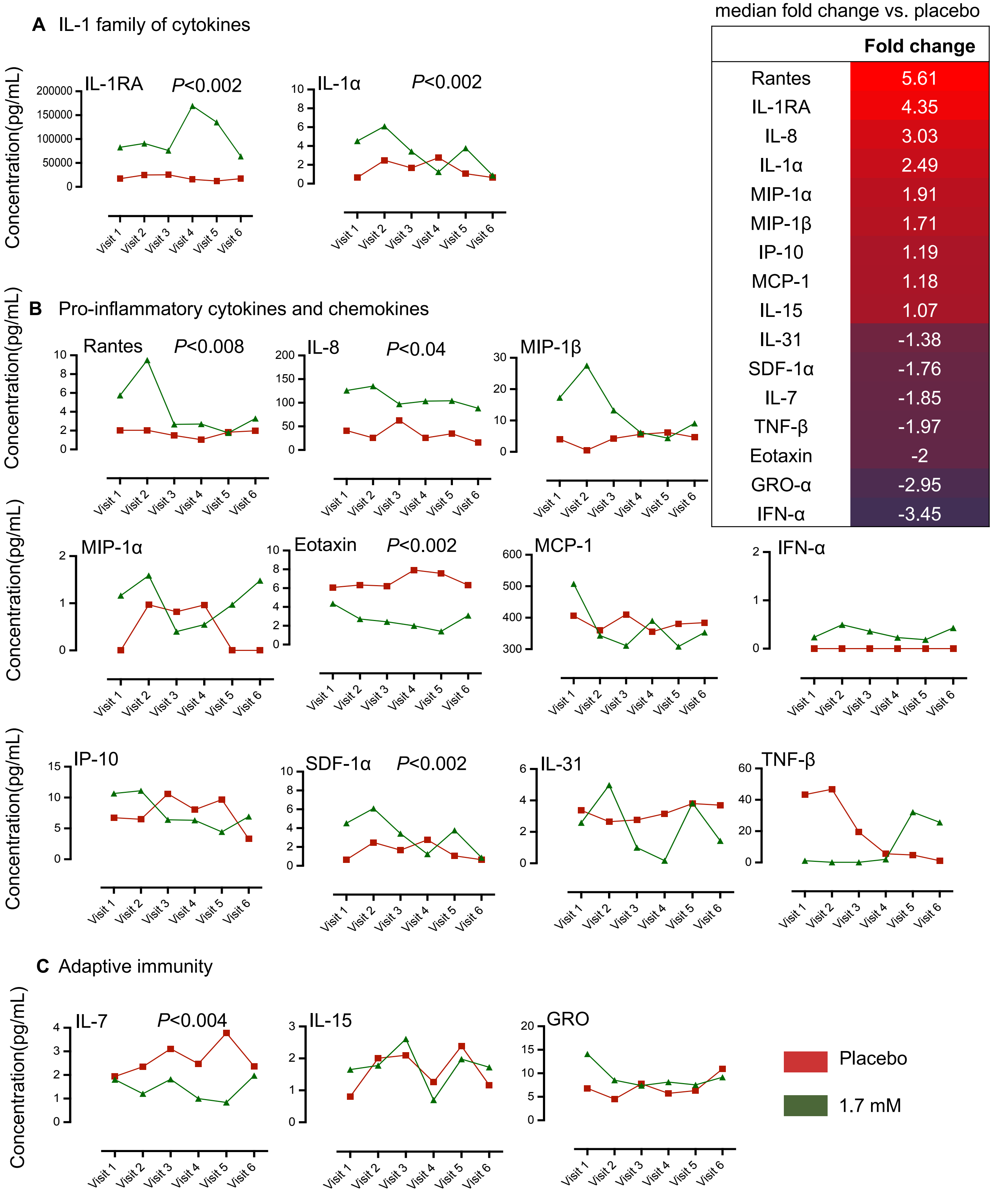

	Fold change
Rantes	5.61
IL-1RA	4.35
IL-8	3.03
IL-1α	2.49
MIP-1α	1.91
MIP-1β	1.71
IP-10	1.19
MCP-1	1.18
IL-15	1.07
IL-31	-1.38
SDF-1α	-1.76
IL-7	-1.85
TNF-β	-1.97
Eotaxin	-2
GRO-α	-2.95
IFN-α	-3.45

Supplementary Figure 2. Initial urine proteomic screen of samples from patients receiving 1.7 mM of alpha1-oleate or placebo, including 16 proteins (MCP-1, MIP-1α, MIP-1β, IP-10, IFN-α, IL-1α, IL-1RA, IL-8, IL-7, IL-31, IL-15, TNF-β, Eotaxin, SDF-1α, Rantes and GRO). Rapid and sustained activation of IL-1RA and IL-8 was detected. Initial activation followed by a reduction was detected for chemokines Rantes, IL-8, and MIP-1β. Eotaxin and IL-7 were inhibited. The remaining cytokines were not significantly altered. Heatmap summarizing the response in the treatment group compared to placebo. Median values were calculated by including samples from all visits for each cytokine and patient group.

Supplementary Fig. 3

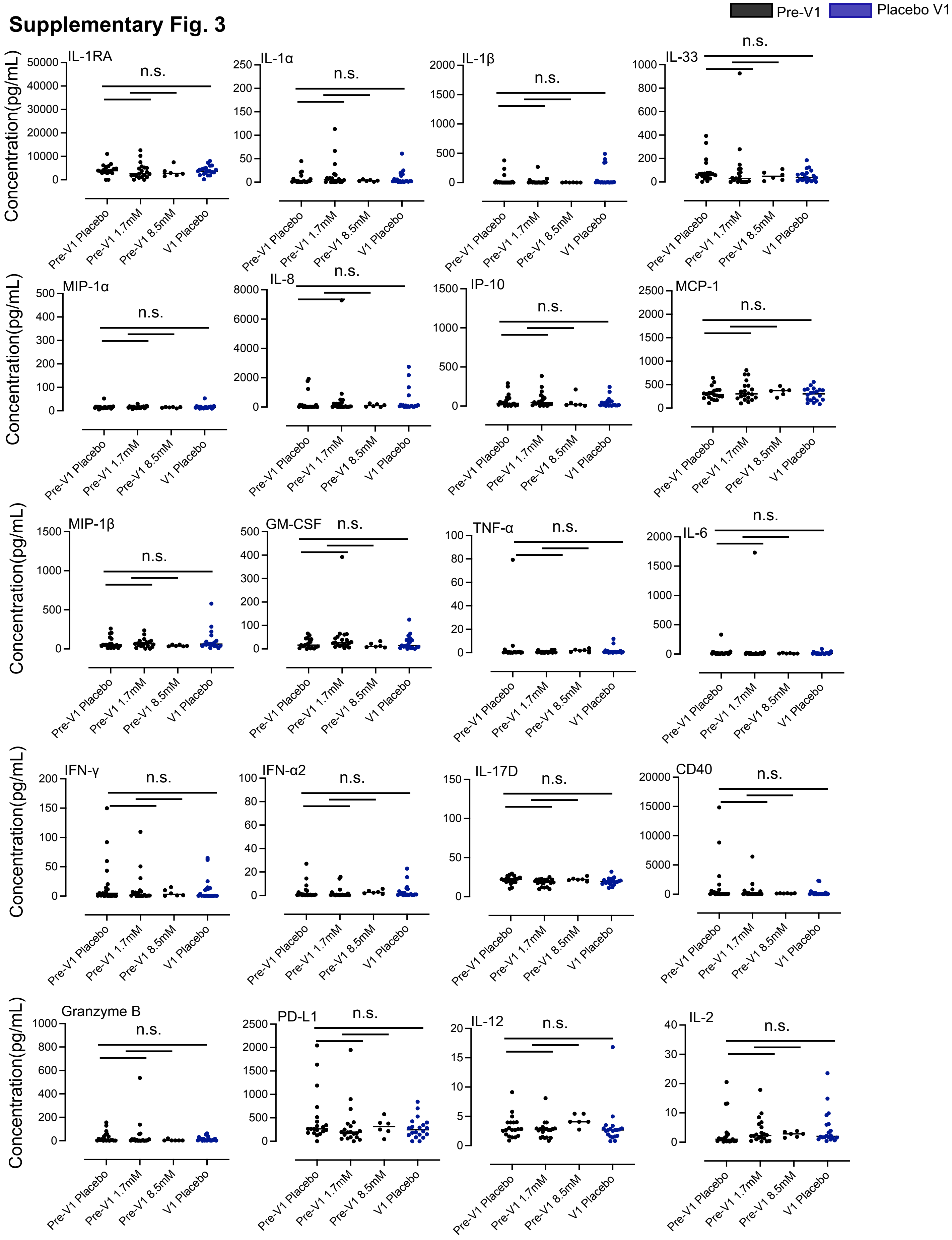

Supplementary Figure 3. Analysis of the urine proteome, comparing the pre-V1 samples to the first post instillation sample in the placebo group. The pre-instillation urine concentrations of the tested cytokines were low and there was no significant difference in protein concentration post installation in the placebo group(Pre-treatment V1= Black, post treatment V1 placebo= Blue) . One Way Anova test, *** *P*<0.001, ** *P*<0.01. * *P*<0.05.

Supplementary Fig. 4 One-Way ANOVA (P<0.05)

Cytokine	8.5 mM vs Placebo	1.7 mM vs Placebo	1.7 mM vs 8.5 mM
IL-1RA	<0.001	<0.001	<0.001
IL-1α	<0.001	<0.001	n.s.
IL-1β	<0.001	0.007	0.03
IL-33	0.03	n.s.	0.002
IL-8	n.s.	n.s.	n.s.
MIP-1α	<0.001	0.004	n.s.
MIP-1β	n.s.	n.s.	n.s.
MCP-1	0.01	n.s.	0.05
GM-CSF	n.s.	n.s.	n.s.
IP-10	<0.001	0.004	n.s.
TNF-α	0.003	n.s.	0.001
IL-6	n.s.	<0.001	<0.001
IFN-γ	<0.001	<0.001	0.02
IFN-α2	<0.001	<0.001	n.s.
CD40	<0.001	0.004	<0.001
PD-L1	0.01	n.s.	0.003
Granzyme B	<0.001	0.002	0.002
IL-17D	<0.001	0.01	0.01
IL-10	n.s.	n.s.	n.s.
IL-2	n.s.	n.s.	0.03
IL-12	0.007	n.s.	<0.001
IL-4	n.s.	n.s.	n.s.
IL-17A	n.s.	n.s.	n.s.
IL-13	n.s.	n.s.	n.s.
IL-15	n.s.	n.s.	n.s.

- *P* < 0.05 significant , n.s. not significant

Supplementary Figure 4. Comparison by One way Anova, of the cytokine response in patients receiving 1.7 mM or 8.5 mM of alpha1-oleate or placebo. Ten cytokines were significantly different in both the 1.7mM and 8.5 mM treatment groups, compared to placebo and five were specific for the 8.5 mM treatment group. *** *P*<0.001, ** *P*<0.01. * *P*<0.05.

Supplementary Fig. 5

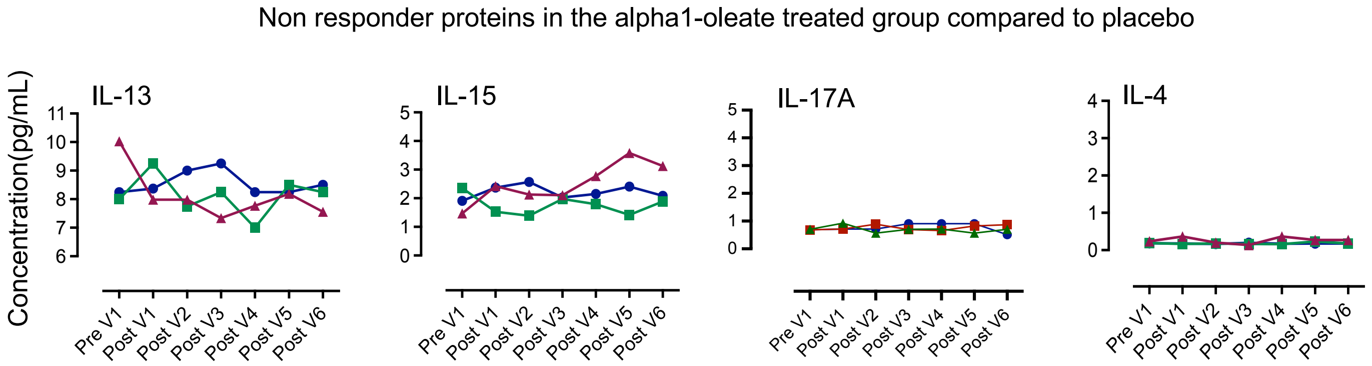

Supplementary Figure 5. Supplementary data for Figure 1. IL-13, IL-15, IL-17A and IL-4 concentrations in urine did not change in response to alpha1-oleate treatment, compared to the placebo group.

Supplementary Fig. 6

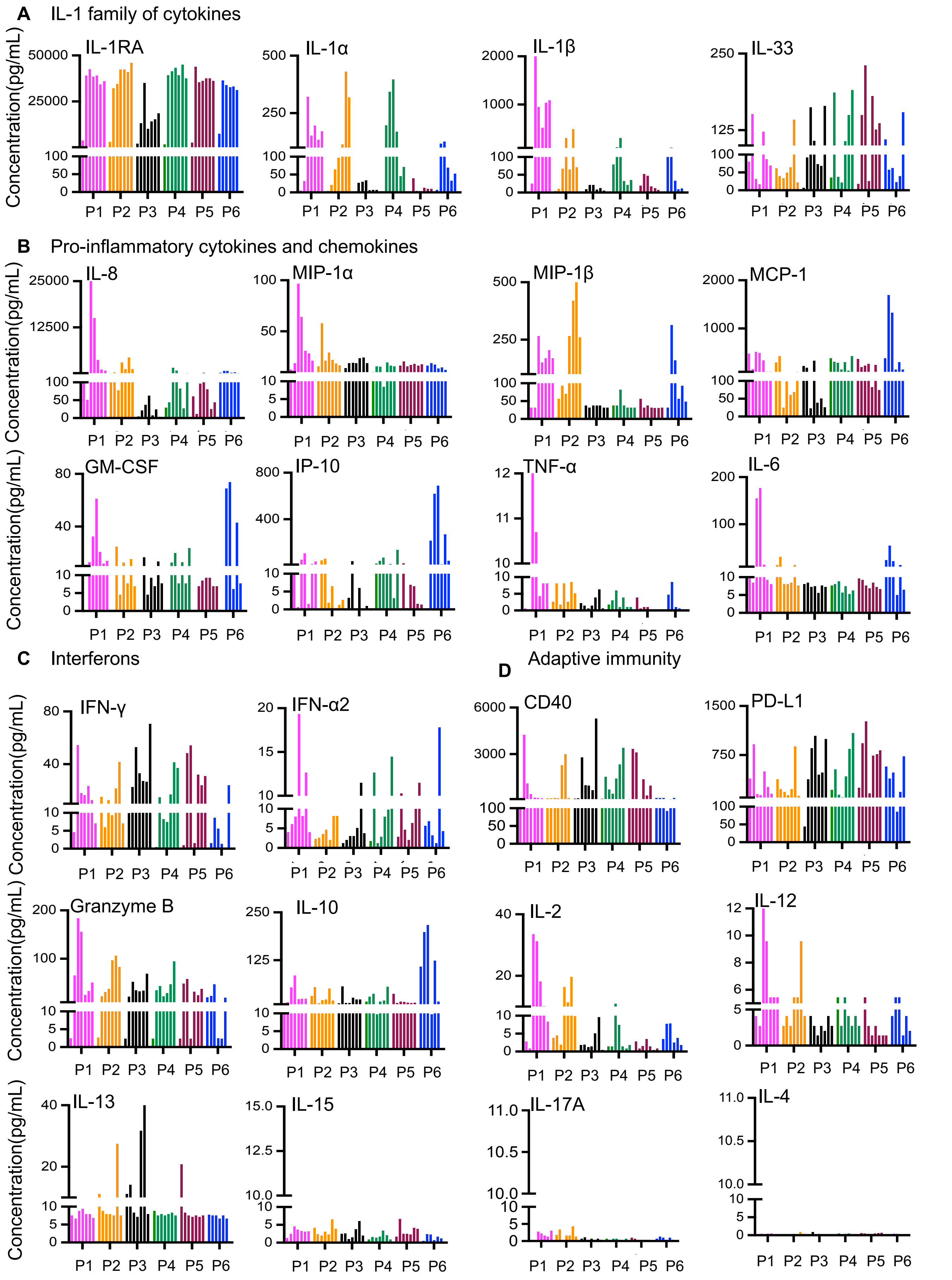

Supplementary Figure 6. Individual patient responses to 8.5 mM alpha1-oleate treatment defined by the median urine protein concentrations after each instillation, compared to the pre-instillation samples obtained at the first visit (pre V1). IL-1RA, IL-1α, IL-1β, IL-33, IL-8, MIP-1α, MIP-1β TNF-α, IFN-α, IFN- γ, Granzyme B, CD40, PD-L1, IL-2, and IL-12, increased significantly after each instillation.

Supplementary Fig. 7

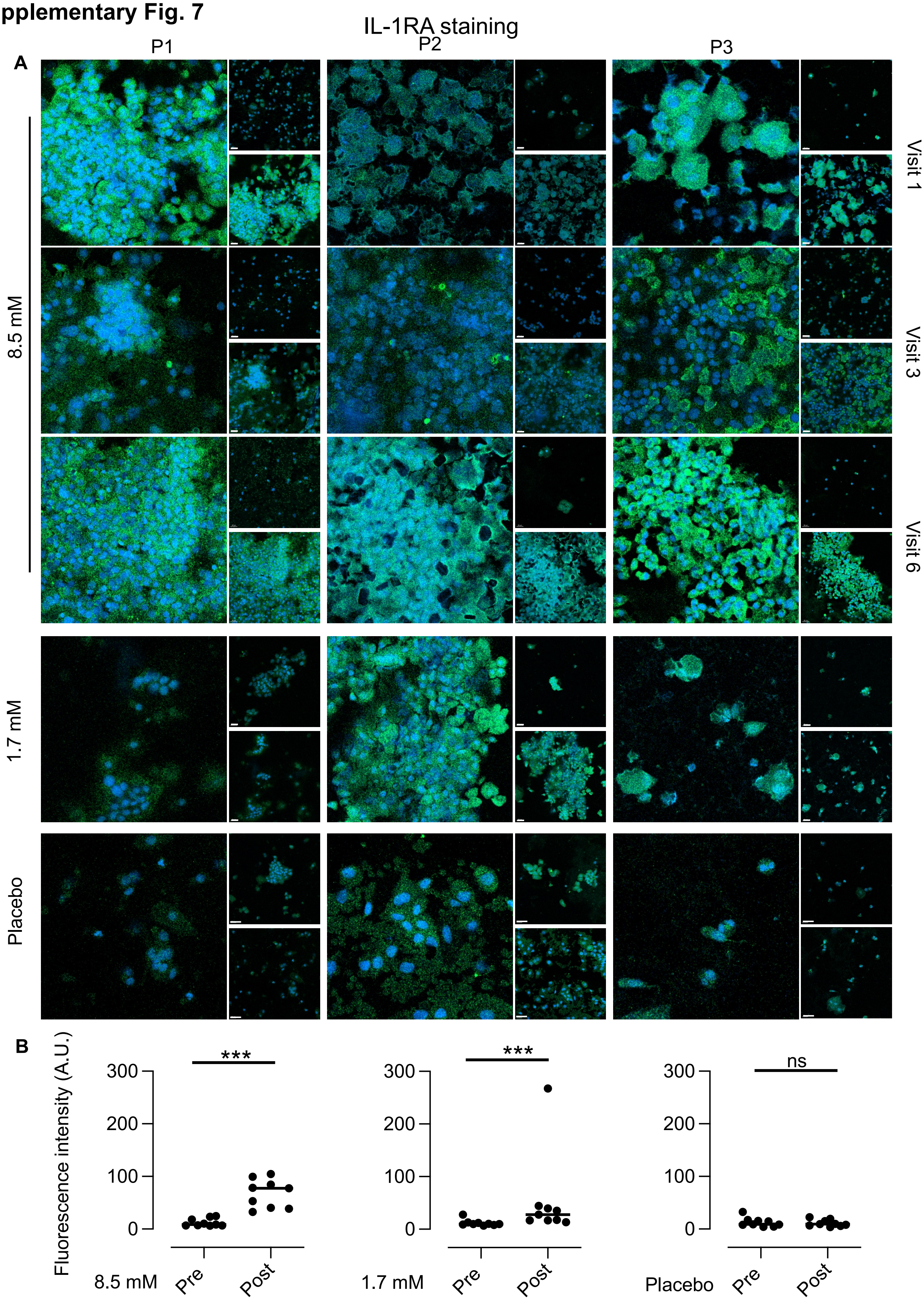

Supplementary Figure 7. IL-1RA response to instillations of alpha1-oleate. Cytokine levels were monitored by immunohistochemistry in the shed cells in patient urine. (**A**) Representative images show a rapid increase in cellular IL-1RA after alpha1-oleate instillation, compared to the pre-instillation sample in each patient. A significant dose dependent increase was also noticed in alpha1-oleate treated patients compared to the placebo group. (3 visits per patient in the 8.5 mM treated group, representative images for the 1.7 mM or placebo group. IL1RA (green), Nuclei (blue). Scale bar 30 µm. (**B**) Comparison of samples obtained pre- or post treatment is shown. Fluorescence intensity was quantified by ImageJ.

Supplementary Fig. 8

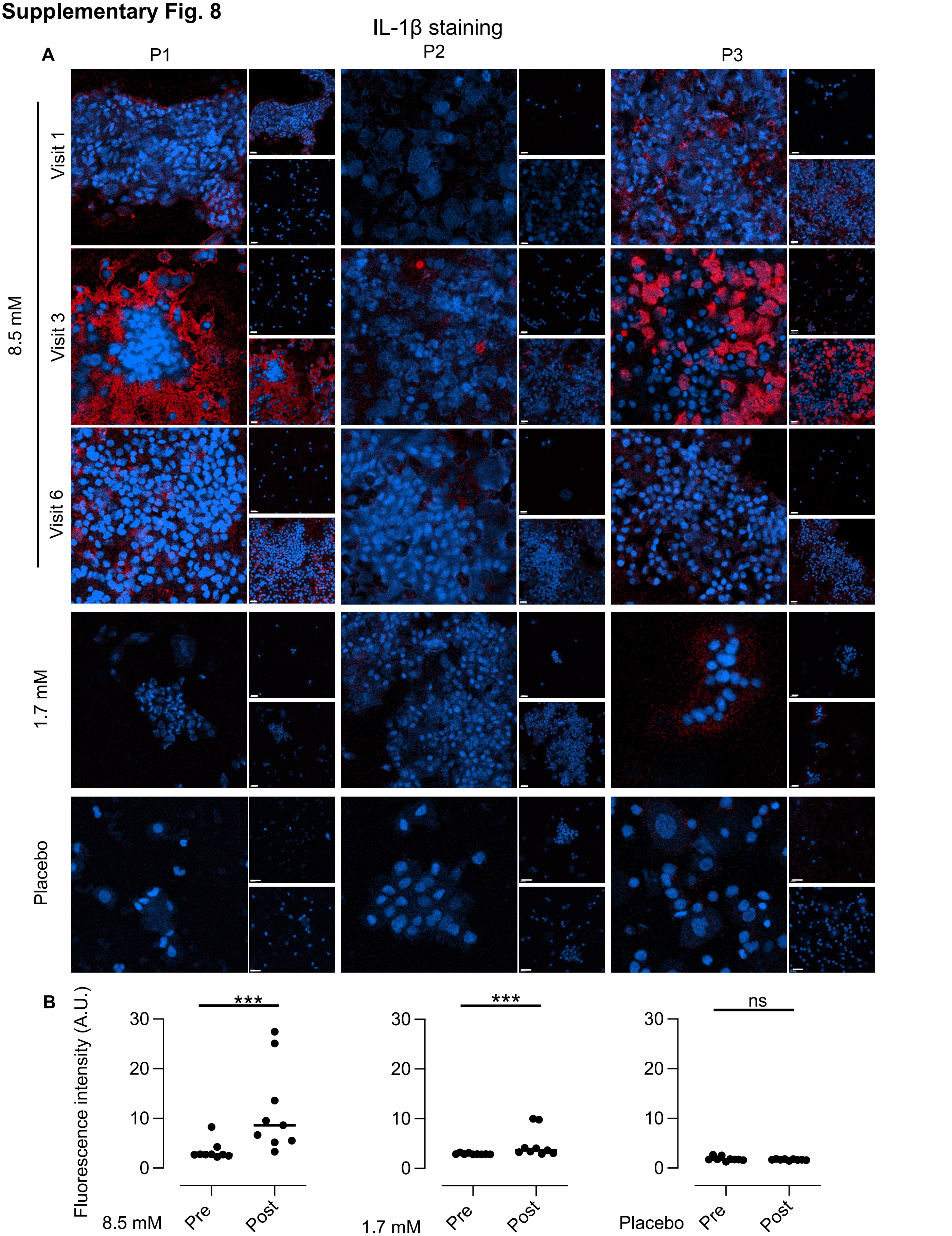

Supplementary Figure 8. IL-1β response to alpha1-oleate instillations. Cytokine levels were monitored by immunohistochemistry of cells in patient urine. (**A**) Representative images show a rapid increase in cellular IL-1β staining after instillations of alpha1-oleate (8.5 mM), compared to the pre-instillation sample in each patient. A significant dose dependent increase was also noticed in alpha1-oleate treated patients, compared to the placebo group. 3 visits per patient for the 8.5 mM treated group, followed by representative images for 1.7 mM and placebo. IL-1β (red), nuclei (blue). Scale bar 30 μm. (**B**) Quantification between pre- and post treatment is shown. Fluorescence intensity was quantified by ImageJ.

Supplementary Fig. 9

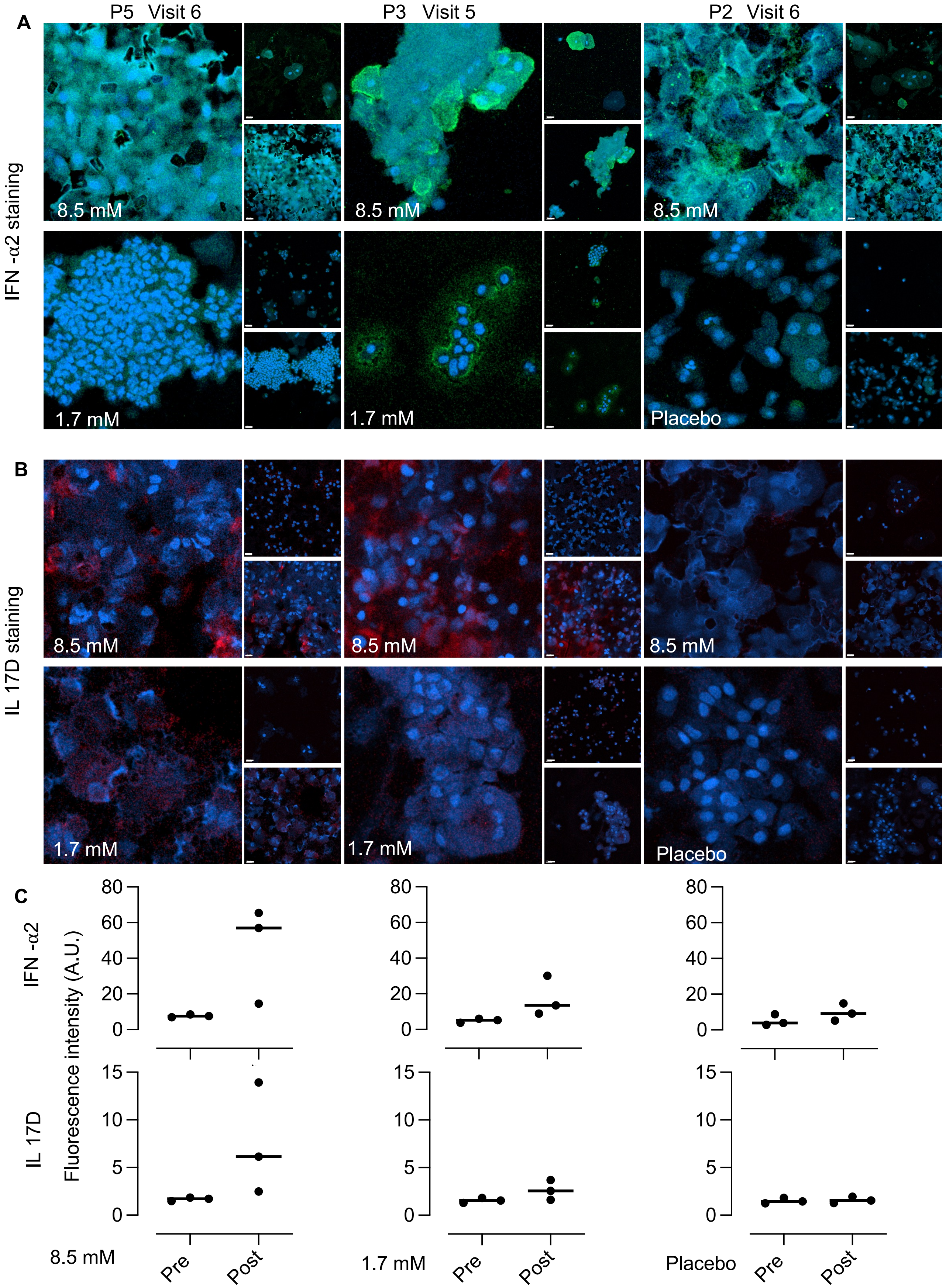

Supplementary Figure 9. IFN-α2, IL17D response to instillations of alpha1-oleate monitored by immunohistochemistry in the cells shed in patient urine. (**A-B**) Representative images show a rapid increase in cellular IFN-α2 staining (A) and cellular IL17D staining (B) after instillation of 8.5 mM of alpha1-oleate, compared to the pre-instillation sample in each patient. A significant dose dependent increase was also noticed in alpha1-oleate treated patients compared to the placebo group. IFN-α2 (green), IL17D (red), Nuclei (blue). 1 visit per patient in the 8.5 mM treated group, followed by representative images for 1.7 mM and placebo. Scale bar 20 µm. (**C**) Quantification between pre and post with each treatment group in shown. Fluorescence intensity was quantified by ImageJ. Mann Whitney test, * P<0.05.

Supplementary Fig. 10

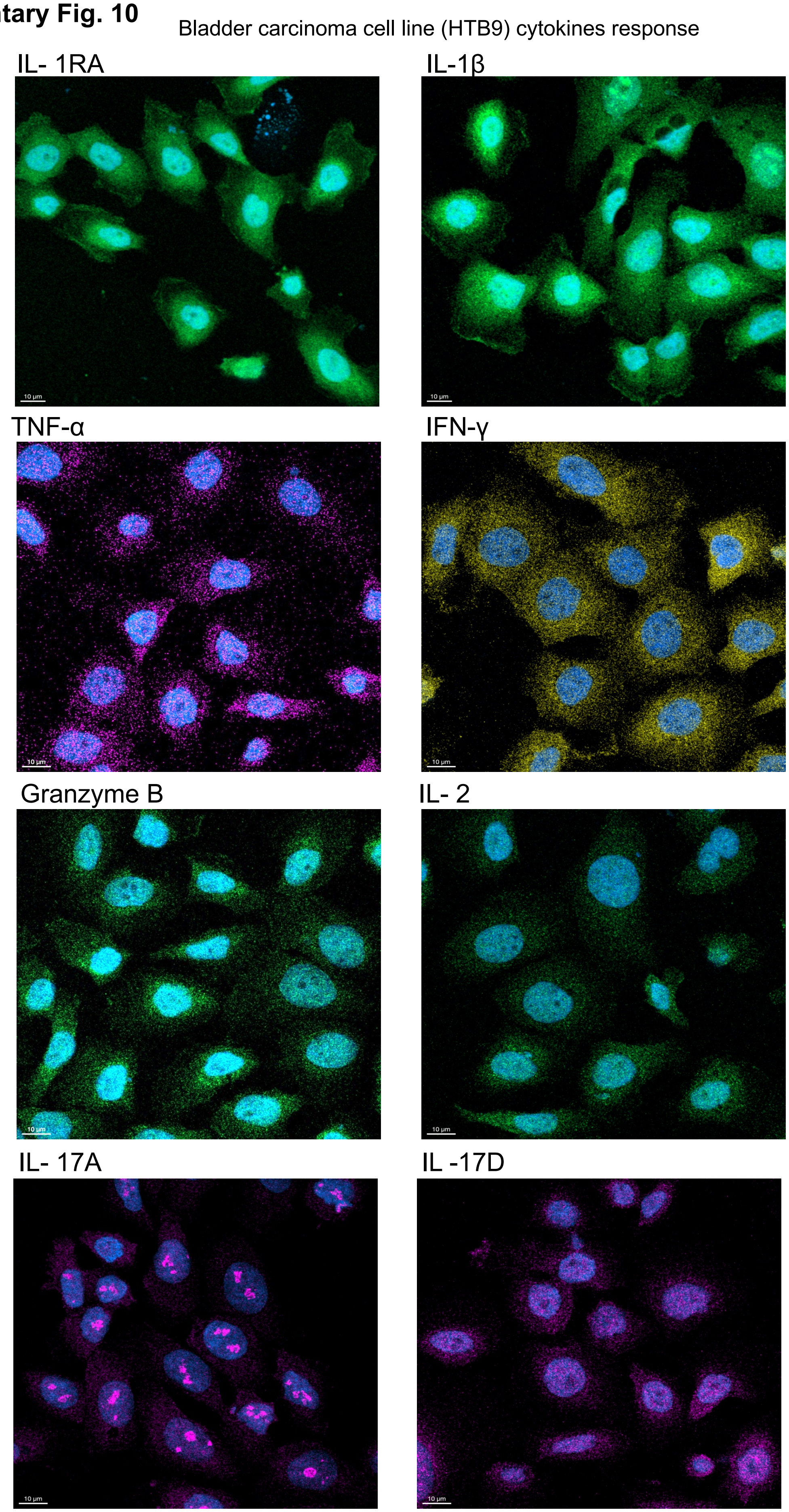

Supplementary Figure 10. Supplementary data for Figure 3. . Cytokine content in human bladder carcinoma cells (HTB9) investigated by immuno-histochemistry, using specific antibodies. HTB9 cells were cultured on microscope slides, fixed and stained with specific antibodies. Imaging was by confocal LSM microscope. The bladder carcinoma cells were shown to contain IL-1RA (green), IL-1β (green), TNF-α (magenta), IFN-γ (yellow), Granzyme B (green), IL-2 (green), IL-17A (magenta) and IL-17D (magenta). Scale bar 10 μm. nuclear staining (blue), Hoechst. Scale bar 10 μm

Supplementary Fig. 11

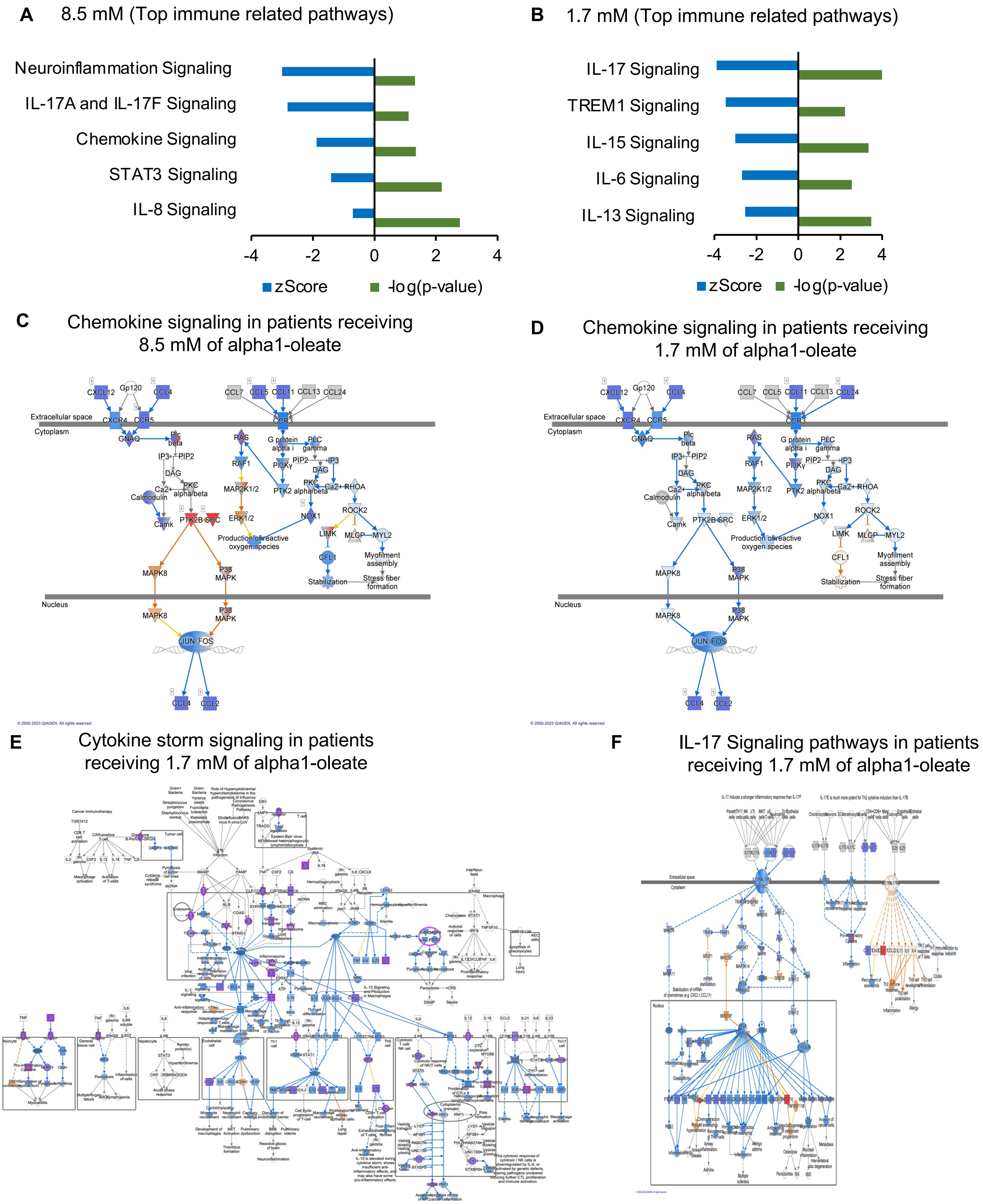

Supplementary Figure 11. (**A-B**) Top immune related pathways in tumors treated with alpha1-oleate compared to placebo (-log(P-value) >1.3, z-score ≤ -2). (**A**) Tumors treated with 8.5 mM of alpha1-oleate showed the downregulation of Neuroinflammation signaling, IL-17A and IL-17F, IL-8, STAT3 and chemokine signaling pathways. (**B**) Tumors treated with 1.7 mM of alpha1-oleate showed the downregulation of IL-17, TREM1, IL-15, IL-6 and IL-13 signaling pathways. (**C-D**). Inhibition of chemokine signaling in tumors treated with 8.5 mM or 1.7 mM of alpha1-oleate (blue = downregulation, red = upregulation, orange = predicted inhibition). (**E-F**) Regulation of canonical pathways in tumor tissues from patients receiving 1.7 mM of alpha1-oleate, compared to the placebo (Blue = downregulation, Red = upregulation). (**E**) Downregulation of the cytokine storm signaling pathway. (**F**) Downregulation of genes in the IL-17 signaling pathway.

Supplementary Fig. 12

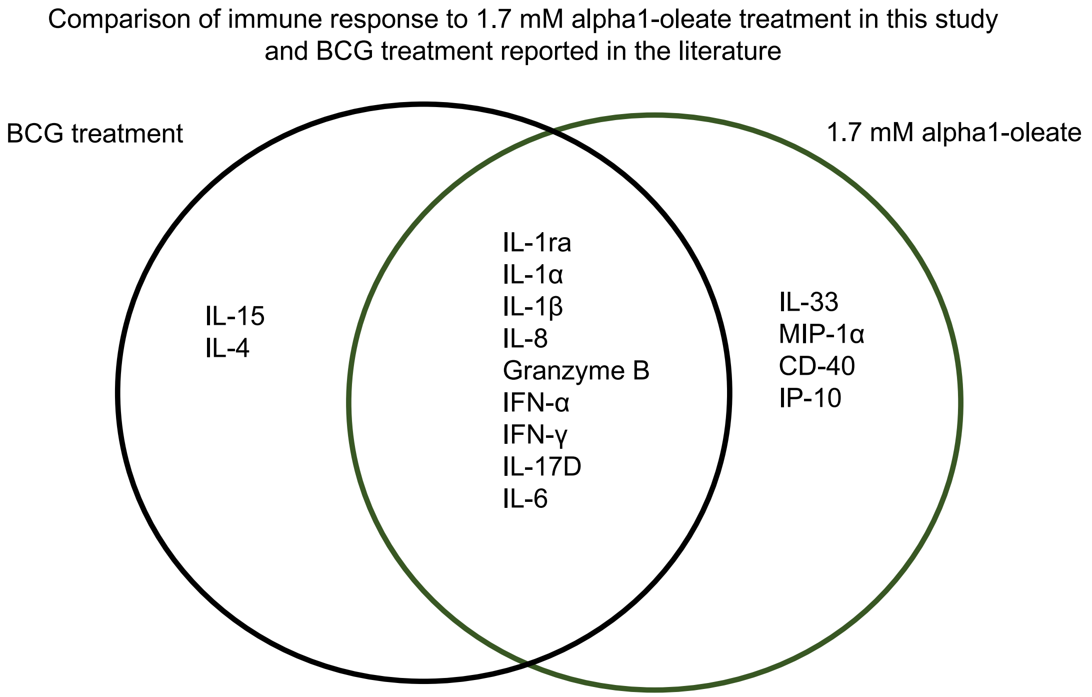

Supplementary Figure 12. Comparative analysis of the alpha1-oleate response in this study and the response to BCG treatment, reported in the literature. Venn diagram visualizing the overlap in cytokine response profiles between patients treated with alpha1-oleate (1.7 mM) in this study and BCG treated patients, reported in the literature. References are shown the legend to Figure 6.

Supplementary Fig. 13

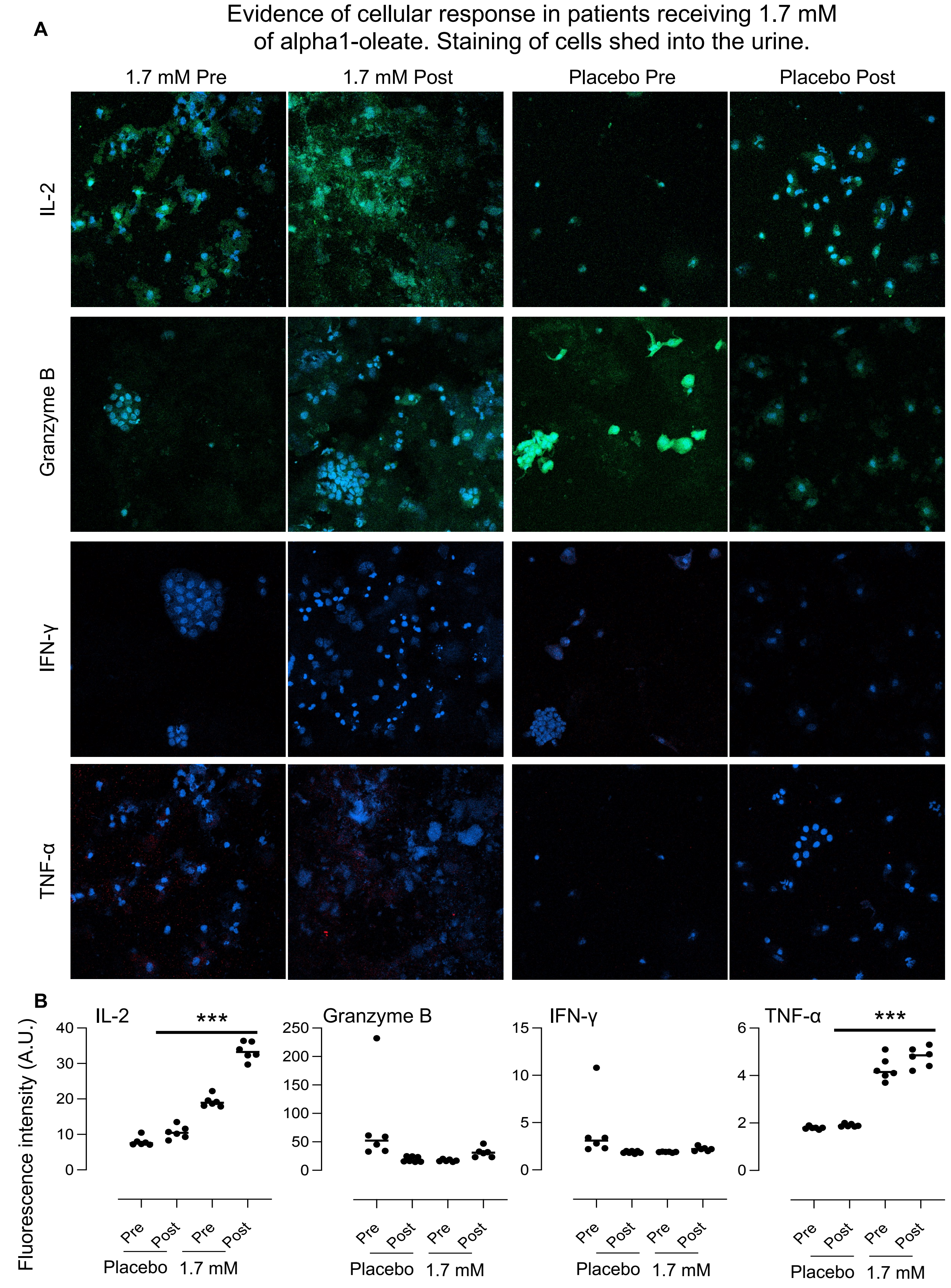

Supplementary Figure 13. (**A**) Cellular response to instillations of alpha1-oleate (IL-2 , Granzyme B, IFN-γ and TNF-α). Urine cytospin cells was examined by immunohistochemistry, using antibodies specific for different cytokines including IL-2, Granzyme B, IFN-γ, and TNF-α. Variable patterns were observed in the 1.7 mM alpha1-oleate treatment group compared to the placebo group. IL-2 , Granzyme B (green); IFN-γ, TNF-α (red); Nuclei (blue). (**B**) Comparisons of the pre -and post treatment groups are shown. Fluorescence intensity was quantified by ImageJ. Mann Whitney test, *** *P*<0.001.

Supplementary Table 1

	CD40		GM-CSF		Granzyme B		IFN-α2		IFN-γ		IL-1α	
	Mean ± SD	Median	Mean ± SD	Median	Mean ± SD	Median	Mean ± SD	Median	Mean ± SD	Median	Mean ± SD	Median
**Placebo**	300.15 ± 612.8	51.8	26.57 ± 28.30	17.49	13.28 ± 19.30	6.06	3.78 ± 5.45	0.93	10.27 ± 15.88	0.85	9.80 ± 17.74	2.63
**1.7 mM**	812.72 ± 1369.57	157	28.67± 43.73	13.69	47.12 ± 102.24	16.74	7.53 ± 11.62	4.37	19.28 ± 26.01	9.21	66.49 ± 110.59	36.79
**8.5 mM**	1228.43 ± 1386.82	667.14	17.55 ± 17.68	11.7	48.68 ± 39.45	35.24	6.93 ± 4.73	5.59	23.49 ± 17.77	21.56	110.02 ± 120.38	64.3

	IL-1β		IL-1RA		IL-2		IL-4		IL-6		IL-8	
	Mean ± SD	Median	Mean ± SD	Median	Mean ± SD	Median	Mean ± SD	Median	Mean ± SD	Median	Mean ± SD	Median
**Placebo**	104.86 ± 287.89	4.15	4118 ± 2209	3951	4.58 ± 5.98	1.86	0.43 ± 0.35	0.18	22.98 ± 70.82	11.07	468.8 ±1024.88	87.52
**1.7 mM**	310.35 ± 1757.54	19.17	10713 ± 8934	10252	3.72 ± 4.73	1.97	0.43 ± 0.36	0.19	21.32 ± 61.69	1.19	734.88 ±1932.81	120.335
**8.5 mM**	223.37 ± 437.65	35.08	34625 ± 9262	36365	6.91 ± 8.23	3.49	0.30 ± 0.21	0.27	19.53 ±3 7.86	8.08	1761.21± 4834.37	198.47

	IL-10		IL-12p-70		IL-13		IL-15		IL-17A		IL-33	
	Mean ± SD	Median	Mean ± SD	Median	Mean ± SD	Median	Mean ± SD	Median	Mean ± SD	Median	Mean ± SD	Median
**Placebo**	44.87 ± 45.79	24.84	2.98 ± 2.42	2.68	9.63 ± 7.18	8.5	3.29 ± 3.50	2.06	5.97± 21.36	0.83	59.48± 55.84	37.02
**1.7 mM**	39.35 ± 41.02	26.4	2.54 ± 1.26	2.81	8.22 ± 4.25	7.63	2.32 ± 2.14	1.73	1.39 ± 2.98	0.6	68.73 ± 65.92	53.5
**8.5 mM**	39.44 ± 48.20	22.19	3.99 ± 2.51	2.73	10.12 ± 7.52	7.56	2.80 ± 1.48	2.49	1.07 ± 0.98	0.71	96.35 ± 60.08	87.01

	IP-10		MCP-1		MIP-1α		MIP-1β		PD-L1		TNF-α	
	Mean ± SD	Median	Mean ± SD	Median	Mean ± SD	Median	Mean ± SD	Median	Mean ± SD	Median	Mean ± SD	Median
**Placebo**	87.37 ± 190.87	51.8	302.92 ± 213.44	271	16.64 ± 9.97	14.3	95.21 ± 86.12	65	283.52 ± 236.05	242	3.93 ± 11.47	0.74
**1.7 mM**	48.40 ± 82.18	157	295.48 ± 184.10	276	24.40 ± 43.82	16.6	124.82 ± 176.30	65	309.85 ± 291.65	219	3.22 ± 14.11	0.81
**8.5 mM**	64.62 ± 156.42	667	269.61 ± 340.73	155	23.54 ± 16.75	19.02	132.73 ± 213.48	38	509.43 ± 349.30	432	3.63 ± 3.57	1.81

Supplementary Table 1. Overview of the urine protein response in 1.7 mM and 8.5 mM alpha1-oleate treated groups compared to the placebo group. Mean ± SD and Median for each cytokine and patient group.
